# Supplementary material for: BICD2 phosphorylation regulates dynein function and centrosome separation in G2 and M
Source: Nat Commun. 2023 Apr 27;14:2434. doi: 10.1038/s41467-023-38116-1 (PMC10140047; doi:10.1038/s41467-023-38116-1)
Supplement: Supplementary file 1 — Supplementary Information [file 41467_2023_38116_MOESM1_ESM.pdf]

BICD2 phosphorylation regulates dynein function and centrosome separation in G2 and M

Núria Gallisà-Suñé[1], Paula Sànchez-Fernàndez-de-Landa[1,§], Fabian Zimmermann[2], Marina Serna[3], Laura Regué[1], Joel Paz[2], Oscar Llorca[3], Jens Lüders[2], Joan Roig[1,*]

[1] Department of Cells and Tissues, Molecular Biology Institute of Barcelona (IBMB-CSIC), Baldiri i Reixac 10-12, 08028 Barcelona, Spain.

[2] Mechanisms of Disease Programme, Institute for Research in Biomedicine (IRB Barcelona), The Barcelona Institute of Science and Technology, Baldiri Reixac 10-12, 08028 Barcelona, Spain.

[3] Structural Biology Programme, Spanish National Cancer Research Centre (CNIO), Melchor Fernández Almagro 3, E-28029 Madrid, Spain.

[§] Present address: Aging and Metabolism Programme, IRB Barcelona.

* Correspondence: jrabmc@ibmb.csic.es

Supplementary Information

This PDF file includes:

Supplementary Figures S1 to S9
Supplementary Methods

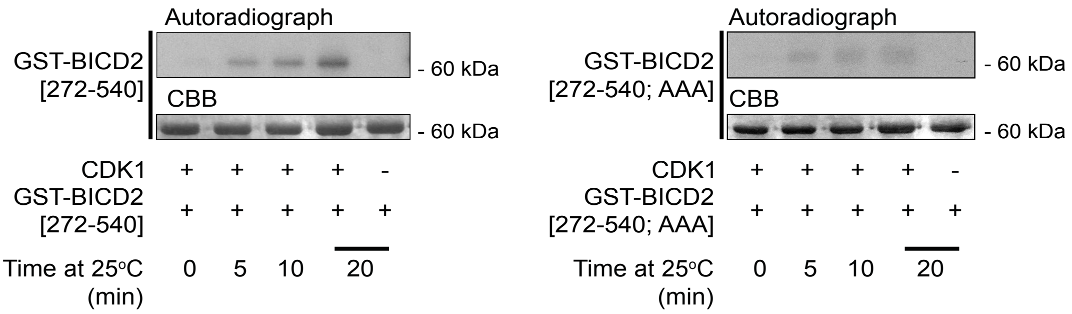

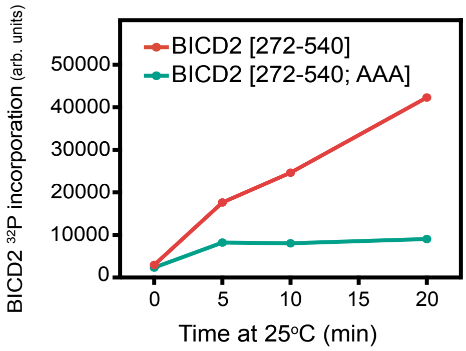

Supplementary Figure S1. Mutation of Thr319, Ser320 and Thr321 strongly interferes with CDK1 phosphorylation of GST-BICD2 [272-541] (*related to Figure 2*).

GST-BICD2 [272-541] and GST-BICD2 [272-541; T319A, S320A, T321A] (*BICD2 [272-541; AAA]*) were incubated with recombinant CDK1/cyclin B plus [γ-^{32}P]ATP/Mg^{2+} at 25°C for the indicated times. One out of two similar experiments is shown. Visualization and quantification as in Figure 2 A.

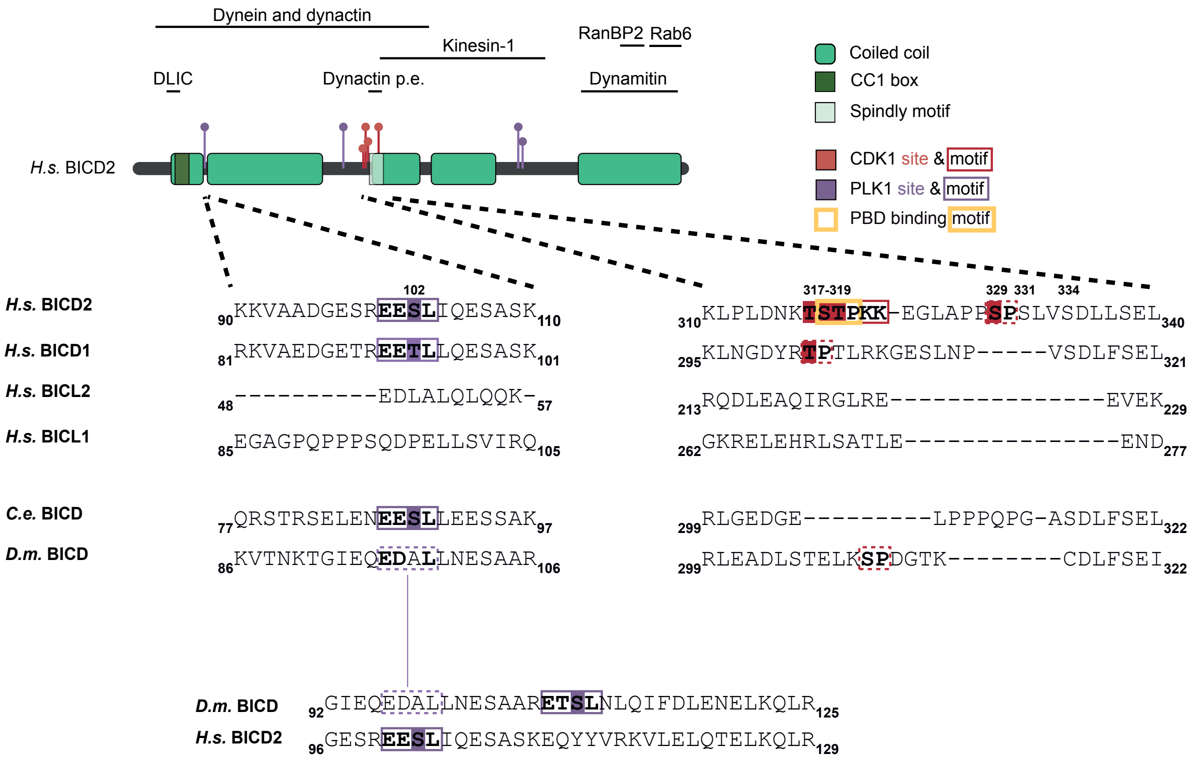

Supplementary Figure S2. Conservation of different BICD2 motifs (*related to Figure 1 and 2*).

Top, schematic representation of BICD2, noting regions interacting with different proteins or protein complexes (*Dynactin p.e., dynactin pointed end)*. Coiled-coil regions have been predicted using paircoil2 [71]. *Bottom,* sequence alignments of residues surrounding Ser102, Thr317, Ser318, Thr319 and Ser329 in different human (*H.s.*), *Caenorhabditis elegans* (*C.e.*) and *Drosophila melanogaster* (*D.m.*) BICD family members. Note that although an orthologue of mammalian Ser102 is not conserved in *Drosophila*, a putative PLK1 phosphorylation site lays ~10 residues to the C-terminus of the protein.

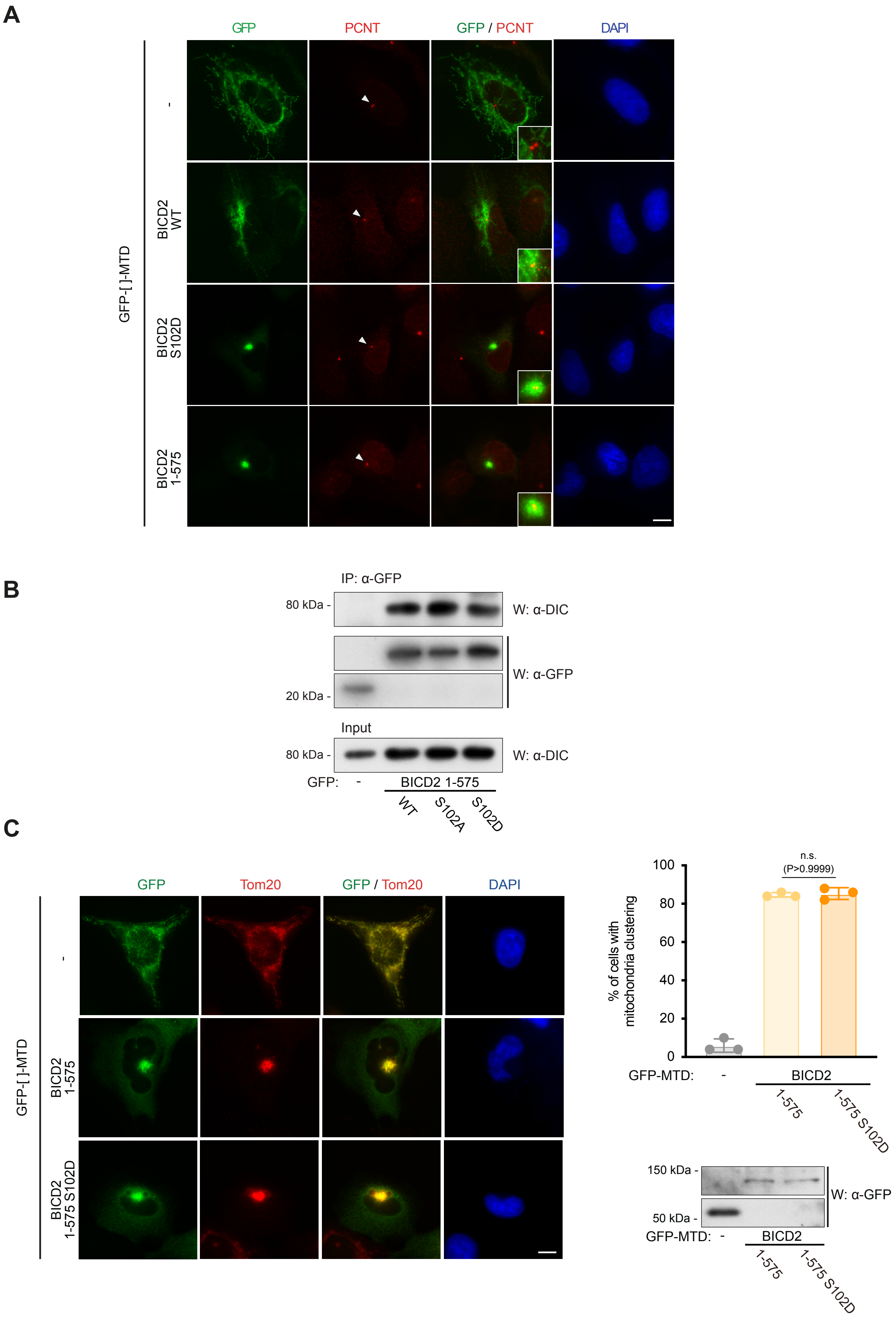
A
GFP
PCNT
GFP / PCNT
DAPI
GFP-[ ]-MTD
-
BICD2 WT
BICD2 S102D
BICD2 1-575
B
IP: α-GFP
80 kDa -
W: α-DIC
W: α-GFP
20 kDa -
Input
80 kDa -
W: α-DIC
GFP:
-
BICD2 1-575
WT
S102A
S102D
C
GFP
Tom20
GFP / Tom20
DAPI
GFP-[ ]-MTD
-
BICD2 1-575
BICD2 1-575 S102D
% of cells with mitochondria clustering
100
80
60
40
20
0
n.s.
(P>0.9999)
GFP-MTD:
-
BICD2
1-575
1-575 S102D
150 kDa -
50 kDa -
W: α-GFP
GFP-MTD:
-
BICD2
1-575
1-575 S102D

Supplementary Figure S3 (*related to Figure 3*).

(A) Mitochondria-targeting domain (MTD)-fused BICD2 constructs cluster mitochondria around centrosomes. The different GFP- and MTD-tagged constructs used in Figure 2B were transfected in U2OS cells. Immunofluorescent staining was done using anti-GFP and anti-pericentrin (used as a centrosomal marker) antibodies, plus DAPI. Scale bar, 10 µm.

(B) Phosphonull or phosphomimetic mutations in Ser102 do not affect dynein binding to BICD2 [1-575]. The indicated GFP-fusion proteins were immunoprecipitated from HeLa cells, and analyzed by western blot to detect dynein using anti-dynein intermediate chain (*DIC*), plus GFP. DIC levels in the corresponding extracts are shown in the lower panel.

(C) A phosphomimetic mutation in BICD2 Ser102 (BICD2 S102D) does not affect dynein mobility towards the centrosomes as detected by mitochondria relocalization and clustering. As in Figure 2B (n=3 biological replicates, 50 cells each; individual replicate means plus mean of replicates ± SD are shown; statistical significance analyzed using a Chi square test). Scale bar, 10 µm. Expression levels of the different polypeptides as detected by western blot are shown.

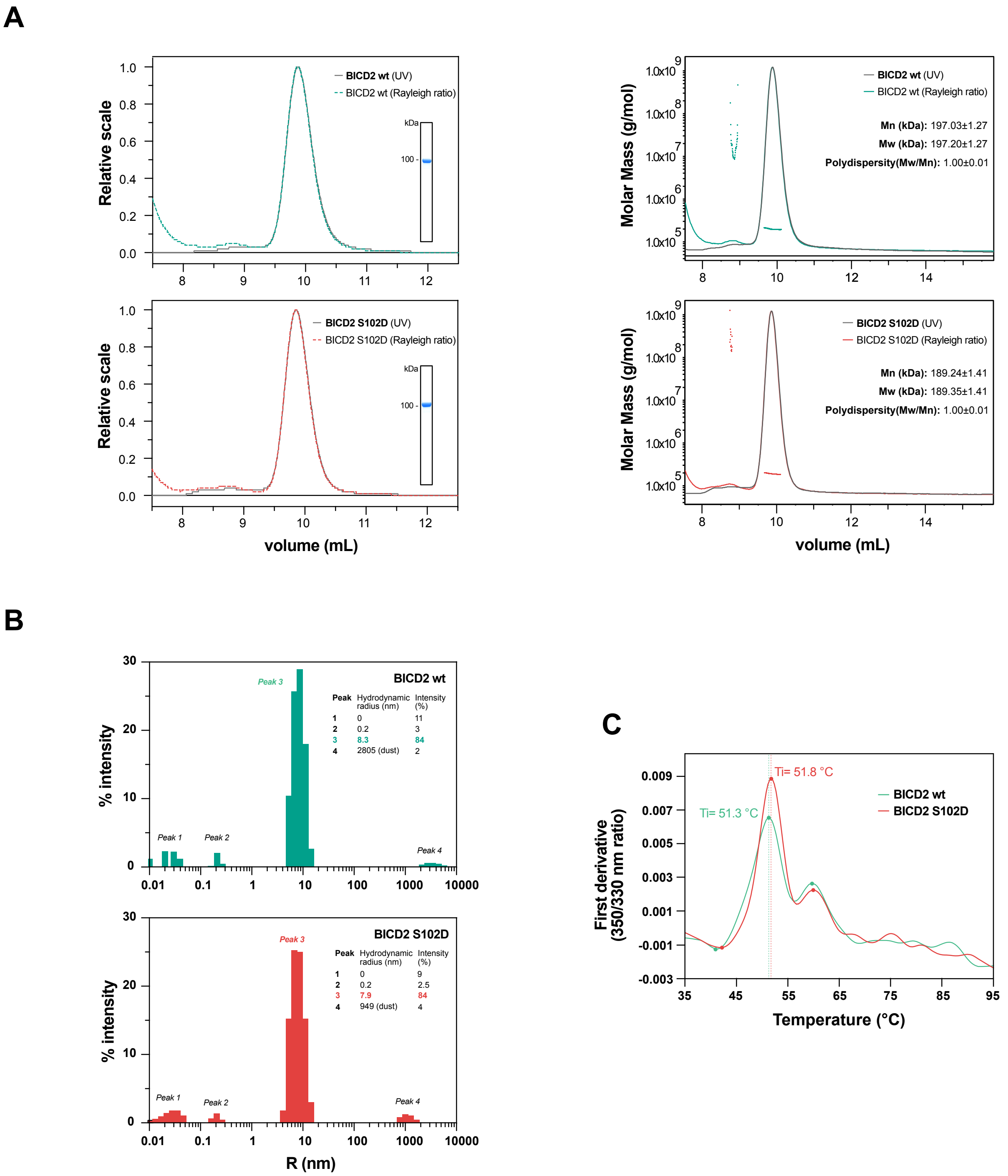

Supplementary Figure S4. SEC-MALS, DLS and denaturation curves for BICD2 wild type and BICD2 S102D (*related to Figure 4*).

(A) SEC-MALS analysis of BICD2 wild type (top) and BICD2 S102D (bottom) performed at 9 µM and 12 µM, respectively. Inlets show Coomassie-stained SDS-PAGE of purified proteins used for analysis. No significant differences in their predicted molecular weight were observed.

(B) Dynamic light scattering (DLS) intensity-based size distribution histograms estimated for BICD2 wildt ype (left panel) and BICD2 S102D (right panel). No significant differences in their hydrodynamic radius (R, nm, in log scale) were observed.

(C) Denaturation curves for BICD2 wild type and BICD2 S102D. Changes in the intrinsic fluorescence of the proteins was measured upon applying a thermal ramp to the sample. The fluorescence signal is plotted as a first derivative of the 350 nm/330 nm ratio. Infection temperature (Ti) is shown.

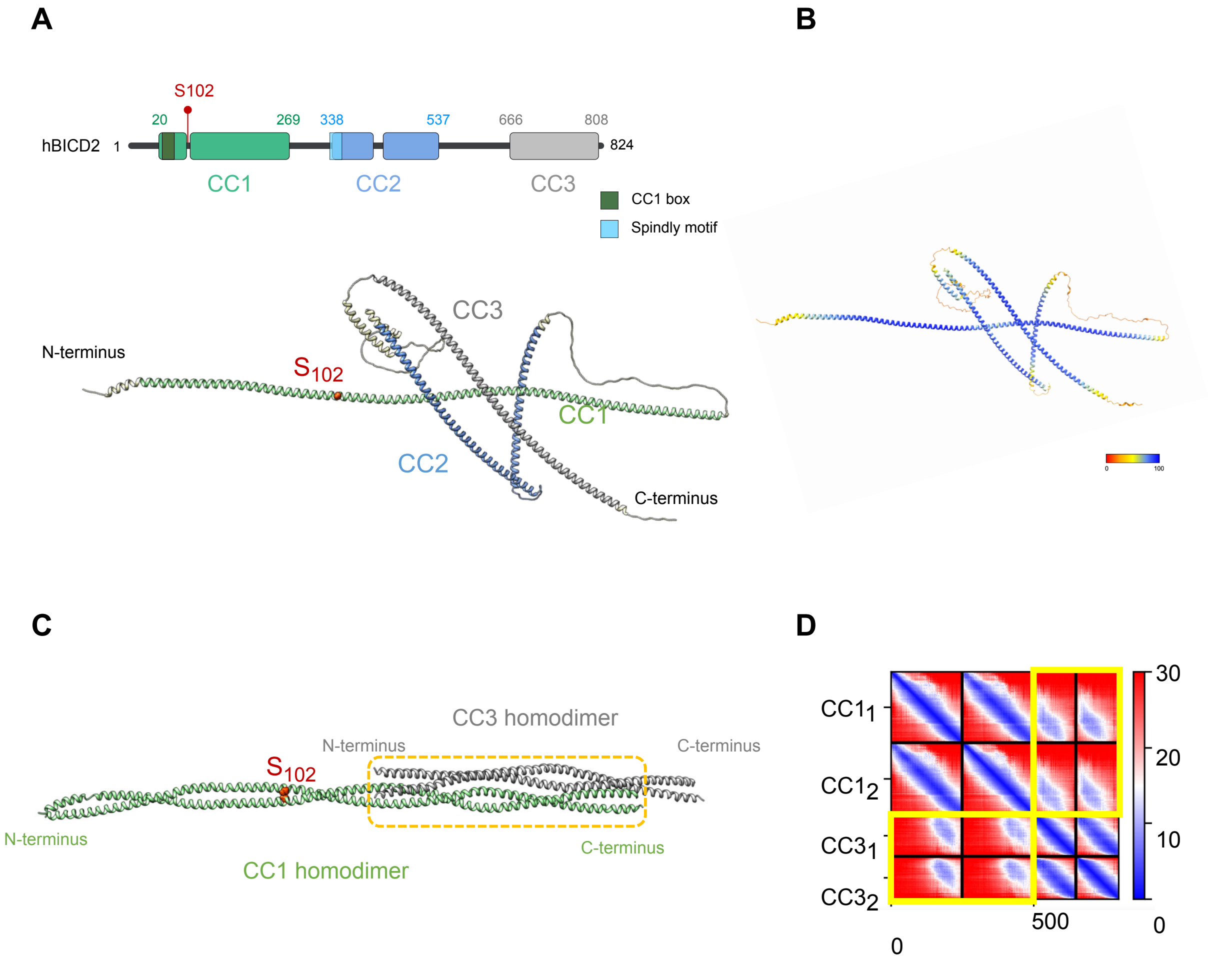

Supplementary Figure S5. Deep learning-based prediction of the human BICD2 atomic model (*related to Figure 4*).

(A) Human BICD2 domain architecture is comprised by three coiled-coil domains named CC1 to CC3 from the N-terminus to the C-terminus. Residue Serine 102 is highlighted in red in both the predicted model and the domains scheme. **(B)** Predicted atomic model of BICD2 coloured based on the pLDDT confidence values measured per residue, ranked from 100 (blue, high accuracy) to 0 (red, low confidence). **(C)** Predicted atomic model of the homodimers BICD2 CC1 and CC3, showing a head-to-head interaction between both coiled-coil homodimers (dashed yellow square). **(D)** Predicted aligned error (PAE) plot of the alphafold model obtained for the homodimers CC1 (CC11 and CC12) and CC3 (CC31 and CC32). This plot represents a range of confidence in the relative domain positions predicted, ranging from low error values (high confidence, in blue) to high error values (low confidence, in red). High confidence relative position is displayed for the CC3 homodimer with the C-terminal end of the CC1 homodimer (yellow squares).

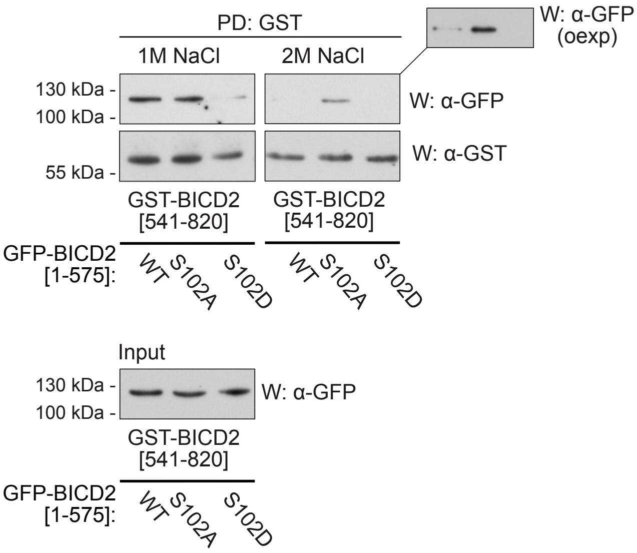

Supplementary Figure S6. Modification of Ser102 interferes with the interaction between BICD2 N- and C-terminal regions (related to Figure 4).

A similar experiment to Figure 4C in which 1M or 2M NaCl was added to the pulldown washing buffer (*oexp*, film overexposure). One out of two similar experiments is shown.

A

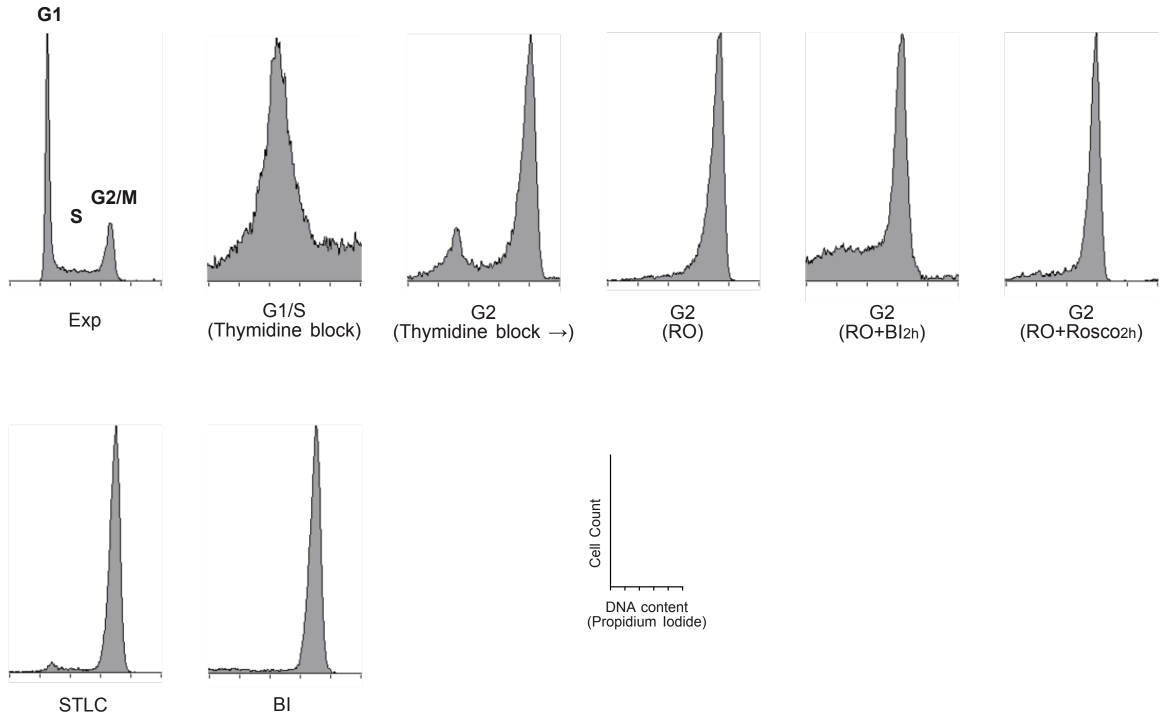

B

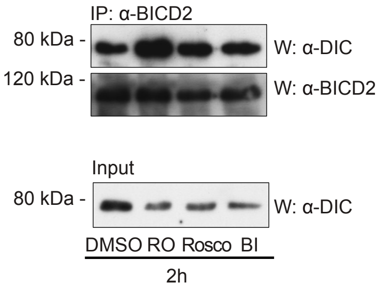

Supplementary Figure S7 (*related to Figure 5*).

(A) DNA content profiles of cells treated as in Figure 5A and B. *Exp*, exponentially growing cells; *G1/S*, cells arrested at the G1/S border after a double thymidine block; *G2*, cells in G2 (*G2*, untreated G2 cells, 8h after being released form a double thymidine block; *G2(RO)*, RO-3306-arrested G2 cells (9 M, 16h); *G2(RO+BI2h)*, RO-3306-arrested G2 cells treated for 2h with 100 nM BI2536;

G2(RO+Rosco2h), RO-3306-arrested G2 cells treated for 2h with 55 μM roscovitine; *STLC*, cells arrested in prometaphase with 5 μM STLC for 16h; *BI*, cells arrested in prometaphase with 100 nM BI2536 for 16h).

(B) Acute CDK or PLK1 inhibition does not significatively disrupts the interaction between BICD2 and dynein in exponentially growing cells. anti-BICD2 immunoprecipitates from exponentially growing HeLa cells treated with the indicated drugs for 2h were analyzed by western blot (*W*) using anti-DIC or anti-BICD2 antibodies. DIC levels in the corresponding extracts are shown in the lower panel. *RO*, 9 μM RO-3306; *Rosco*, 55 μM roscovitine; *BI*, 100 nM BI2536). One out of two similar experiments is shown.

A

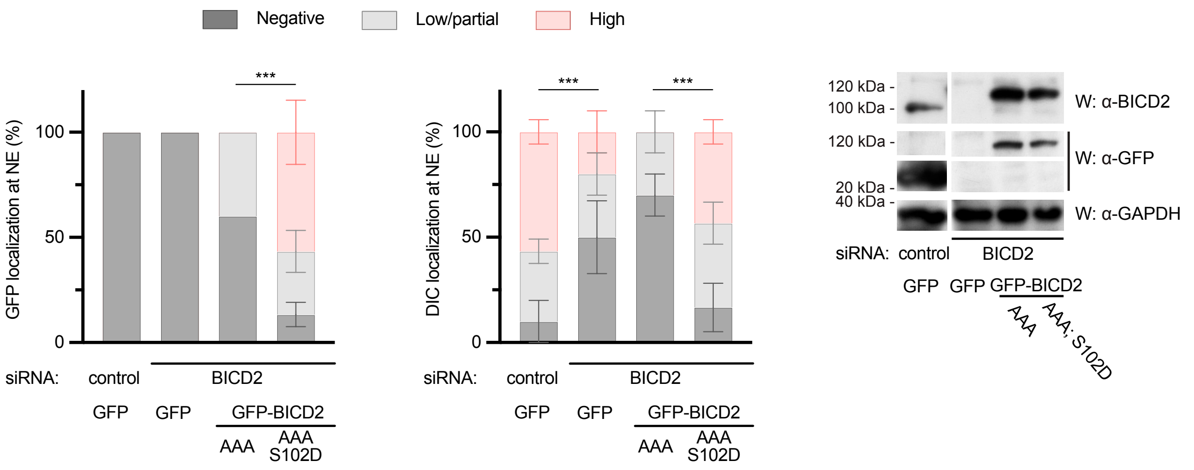

B

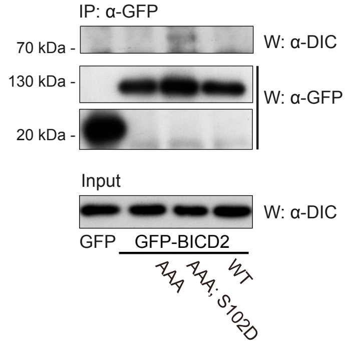

Supplementary Figure S8 (*related to Figure 6*).

(A) A phosphomimetic mutation in BICD2 Ser102 (S102D) is able to rescue dynein nuclear envelope localization in G2 cells with low endogenous BICD2 levels even if major BICD2 CDK1 sites (T319A, S320A, Thr321A) (*BICD2 AAA*) are mutated. HeLa cells were transfected and stained as in Figure 5. GFP-positive G2 cells, identified by positive cyclin B1 cytoplasmic staining, were scored for GFP and DIC at the nuclear envelope and the results quantified as shown (n=3 biological replicates, 10 cells per experiment; mean ± SD is shown; statistical significance was analyzed using a Chi square test with a two-sided P value; *** P<0.001). Expression levels of endogenous BICD2 and GFP-fusion proteins are shown.

(B) The phosphomimetic mutation S102D is able to support dynein binding to BICD2 in a form of the adaptor with mutated BICD2 CDK1 sites (T319A, S320A, T321A). The indicated GFP-fusion proteins were immunoprecipitated from HeLa cells and analyzed by western blot to detect dynein using anti-dynein intermediate chain (DIC) antibodies plus GFP. DIC levels in the corresponding extracts are shown in the lower panel.

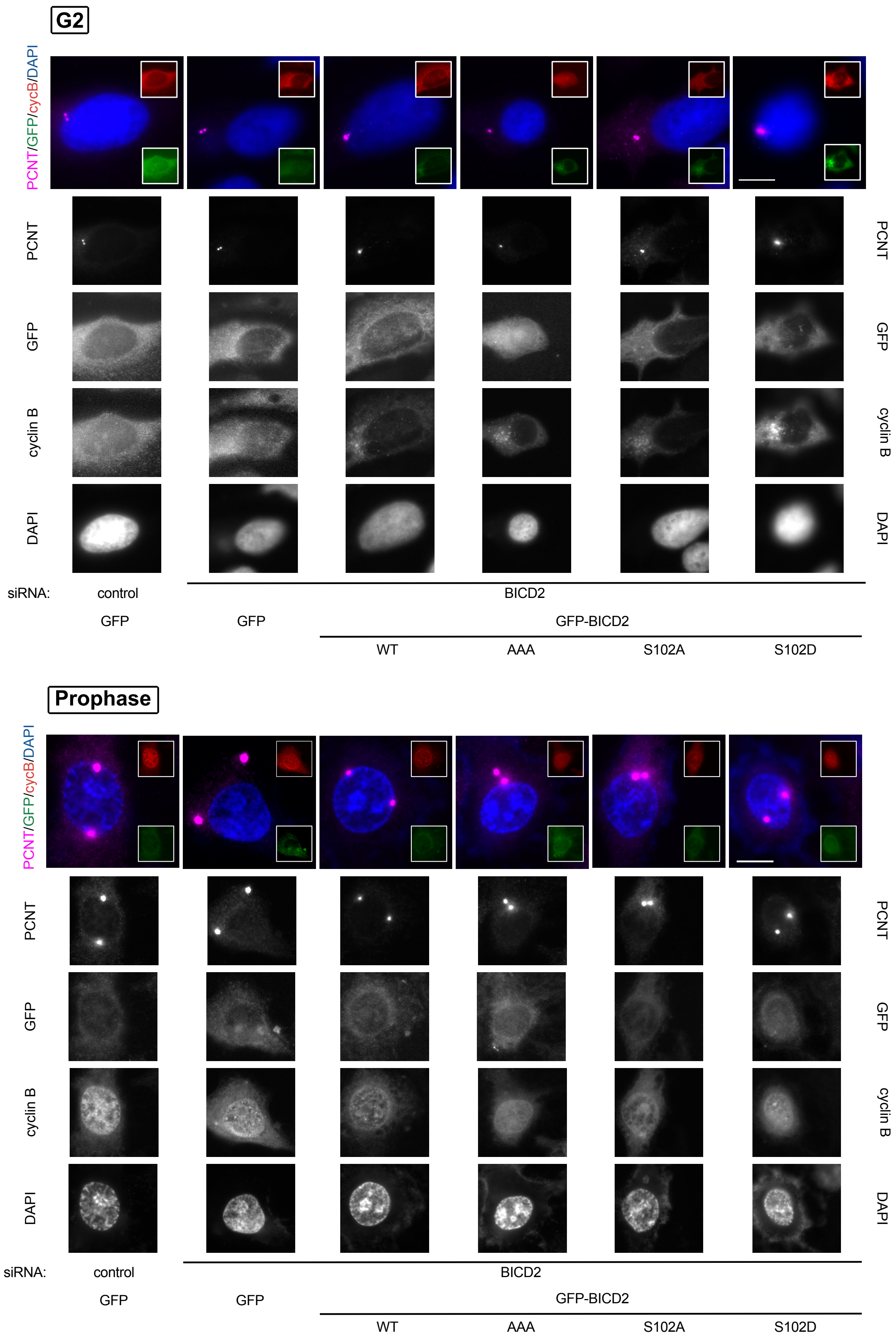

Supplementary Figure S9. Example images in figure 7A and 7D showing the individual light channels (*related to Figure 7*).

Supplementary Methods

SEC-MALS

To determine the molecular weights of purified full-length BICD2 wildtype and S102D, size exclusion chromatography (SEC) combined with light scattering analysis (MALS) was used. SEC data were measured using a Superdex 200 increase 10/300 GL column (GE Healthcare) connected to a HPLC system (Prominence, Shimadzu). The elution from SEC was monitored by an SPD-20 UV detector (Shimadzu), an OPTI-rEx differential refractometer (Wyatt) and a DAWN-HELEOS static MALS detector (Wyatt). Data analysis was performed with ASTRA 7 software (Wyatt).

DLS

BICD2 wildtype and S102D samples were prepared at 0.3 mg/ml in 25 mM HEPES-NaOH pH 7.5, 150 mM NaCl, 1 mM TCEP and 5% glycerol, and centrifuged in a benchtop centrifuged at 14.500 rpm for 15 min. Afterwards, 20 µL of each sample were loaded in a 1.5 mm path length cuvette and subjected to dynamic light scattering (DLS) measurements after ~2 min for equilibration.

All the DLS experiments were conducted on a DynaPro MS/X instrument (Protein Solutions) with a 50 mW laser at 825.3 nm and a temperature controlled microsampler. The detector angle was 90º. The laser power was 100% and each data set corresponds to 20 acquisitions with 10 s acquisition time at 25 ºC. For analysis, only acquisitions fitting to the autocorrelation function with a sum of squares (SOS) error below 100 were considered.

Thermal stability evaluation

Thermal stability of both BICD2 wildtype and S102D mutant were evaluated by measuring the protein intrinsic fluorescence at 330 and 350 nm upon a thermal ramp using a Tycho NT.6 nanotempter instrument. The sample was diluted to 0.5 mg/ml in protein buffer (25 mM Tris-HCl pH 8.0, 300 mM NaCl, 0.5 mM TCEP) and centrifuged in a bench centrifuge for 5 min at maximum speed. Measures were obtained at 25 ºC.
