# Supplementary material for: BICD2 phosphorylation regulates dynein function and centrosome separation in G2 and M
Source: Nat Commun. 2023 Apr 27;14:2434. doi: 10.1038/s41467-023-38116-1 (PMC10140047; doi:10.1038/s41467-023-38116-1)
Supplement: Supplementary file 3 — Reporting Summary [file 41467_2023_38116_MOESM3_ESM.pdf]

nature portfolio

Corresponding author(s): Joan Roig

Last updated by author(s): Mar 28, 2023

Reporting Summary

Nature Portfolio wishes to improve the reproducibility of the work that we publish. This form provides structure for consistency and transparency in reporting. For further information on Nature Portfolio policies, see our Editorial Policies and the Editorial Policy Checklist.

Statistics

For all statistical analyses, confirm that the following items are present in the figure legend, table legend, main text, or Methods section.

n/a	Confirmed	
☐	☒	The exact sample size (*n*) for each experimental group/condition, given as a discrete number and unit of measurement
☐	☒	A statement on whether measurements were taken from distinct samples or whether the same sample was measured repeatedly
☐	☒	The statistical test(s) used AND whether they are one- or two-sided<br>*Only common tests should be described solely by name; describe more complex techniques in the Methods section.*
☒	☐	A description of all covariates tested
☒	☐	A description of any assumptions or corrections, such as tests of normality and adjustment for multiple comparisons
☐	☒	A full description of the statistical parameters including central tendency (e.g. means) or other basic estimates (e.g. regression coefficient) AND variation (e.g. standard deviation) or associated estimates of uncertainty (e.g. confidence intervals)
☐	☒	For null hypothesis testing, the test statistic (e.g. *F*, *t*, *r*) with confidence intervals, effect sizes, degrees of freedom and *P* value noted<br>*Give P values as exact values whenever suitable.*
☒	☐	For Bayesian analysis, information on the choice of priors and Markov chain Monte Carlo settings
☒	☐	For hierarchical and complex designs, identification of the appropriate level for tests and full reporting of outcomes
☒	☐	Estimates of effect sizes (e.g. Cohen's *d*, Pearson's *r*), indicating how they were calculated

Our web collection on statistics for biologists contains articles on many of the points above.

Software and code

Policy information about availability of computer code

Data collection	Three channel Z-stack images were acquired in a Leica AF6000 system with an Orca AG camera (Hamamatsu) coupled to a Leica DMI6000B microscope equipped with 63x/1.40NA and 100x/1.40NA oil immersion lens and the standard LAS-AF software (version 3.0.0. build 81.34) followed with deconvolution. Four channel Z-stack images were acquired with a Leica Thunder system with a DMI8 microscope equipped with 100x NA 1.40 HCX PL-APO oil immersion lens and the standard LASX software (version 3.7.4.23463) followed with deconvolution. Four channel Z-stack confocal images were obtained using a Zeiss Lsm780 confocal system with an inverted XYZ Zeiss Axio Observer Z1 microscope equipped with an 63x/1.40NA oil immersion lens and the standard Zeiss software (ZEN, version 2.1). Images were analyzed using FIJI versions 2.1.0 to 2.9.0.<br><br>Electron microscope images were acquired in a FEI Tecnai G2 Spirit microscope with a Lab6 filament and operated at 120 kV, using a TVIPS CCD camera. Microscope images were processed using the cryoSPARC v3.2 70 software package. Automatically selected particles were aligned and classified using the reference-free 2D classification tool in cryoSPARC.
Data analysis	Data analysis was carried out with Prism (version 9.5.1).

For manuscripts utilizing custom algorithms or software that are central to the research but not yet described in published literature, software must be made available to editors and reviewers. We strongly encourage code deposition in a community repository (e.g. GitHub). See the Nature Portfolio guidelines for submitting code & software for further information.

March 2021

Data

Policy information about availability of data

All manuscripts must include a data availability statement. This statement should provide the following information, where applicable:

- Accession codes, unique identifiers, or web links for publicly available datasets
- A description of any restrictions on data availability
- For clinical datasets or third party data, please ensure that the statement adheres to our policy

The authors declare that all the data supporting the findings of this study are available within the article and its supplementary information files. Sequences in Figure 2I and S2 are from Uniprot (https://www.uniprot.org/ , releases up to 2023_01)

Human research participants

Policy information about studies involving human research participants and Sex and Gender in Research.

Reporting on sex and gender	N/A
Population characteristics	N/A
Recruitment	N/A
Ethics oversight	N/A

Note that full information on the approval of the study protocol must also be provided in the manuscript.

Field-specific reporting

Please select the one below that is the best fit for your research. If you are not sure, read the appropriate sections before making your selection.

☒ Life sciences ☐ Behavioural & social sciences ☐ Ecological, evolutionary & environmental sciences

For a reference copy of the document with all sections, see nature.com/documents/nr-reporting-summary-flat.pdf

Life sciences study design

All studies must disclose on these points even when the disclosure is negative.

Sample size	Sample sizes were determined based on experience from previous work and other similar published studies such as Baffet et al., 2015 (10.1016/j.devcel.2015.04.022).
Data exclusions	No data was excluded.
Replication	Quantitative experiments were replicated two or three times with similar outcome. Non-quantitative experiments at least two times with similar results.
Randomization	Not relevant to the study, as no animal or human subjects were involved in it.
Blinding	Investigators were not blinded to group allocations, as groups were in most experiments easily identified by differences in immunostaining patterns, thus rendering it ineffective.

Reporting for specific materials, systems and methods

We require information from authors about some types of materials, experimental systems and methods used in many studies. Here, indicate whether each material, system or method listed is relevant to your study. If you are not sure if a list item applies to your research, read the appropriate section before selecting a response.

Materials & experimental systems

n/a	Involved in the study
☐	☒ Antibodies
☐	☒ Eukaryotic cell lines
☒	☐ Palaeontology and archaeology
☒	☐ Animals and other organisms
☒	☐ Clinical data
☒	☐ Dual use research of concern

Methods

n/a	Involved in the study
☒	☐ ChIP-seq
☒	☐ Flow cytometry
☒	☐ MRI-based neuroimaging

Antibodies

Antibodies used

a-PLK1 (mouse monoclonal IgG2b, Calbiochem #DR1037; WB, 1:1000)
a-BICD2 (rabbit polyclonal, Abcam #ab117818 for western blot (1:1000) and immunoprecipitations (IP); for immunofluorescence (1:300), rabbit polyclonal #2293, a gift from Anna Akhmanova (Utrecht University), described in 14.)
a-GFP (mouse monoclonal IgG2a, Thermo Fisher #A11120; WB, 1:1000; IF, 1:1000); rabbit polyclonal Torrey Pines #TP401 (IF: 1:500) and Santa Cruz #sc-8334 (IF, 1:500)
a-p150 (mouse monoclonal IgG2b, Santa Cruz #sc-365274; WB, 1:1000)
a-DIC (mouse monoclonal IgG2b, Santa Cruz #sc-13524 for WB (1:1000) and IP; mouse monoclonal IgG2b, Thermo Fisher #14-97772-80 for IF(1:500))
a-GST (mouse monoclonal IgG1, Sigma #SAB4200237; WB: 1:1000)
a-GAPDH (mouse monoclonal IgG1, Santa Cruz #sc-47724; WB: 1:1000)
a-Tom20 (mouse monoclonal IgG2a, Santa Cruz #sc-17764; IF, 1:250)
a-PCNT (rabbit polyclonal, Abcam #ab4448; IF: 1:1000)
a-CycB (mouse monoclonal IgG1, Santa Cruz #sc-245; IF: 1:2000)

Secondary antibodies used were as follows (Alexa Fluor conjugates, Thermo Fisher; HRP conjugates, R&D):
Alexa Fluor 647 goat anti-rabbit IgG (H+L) #A21244 (IF: 1:500-1:1000)
Alexa Fluor 568 goat anti-mouse IgG2b #A21144 (IF: 1:500-1:1000)
Alexa Fluor 488 goat anti-mouse IgG1 #A21121 (IF: 1:500-1:1000)
Alexa Fluor 488 goat anti-rabbit IgG (H+L) #A11008 (IF: 1:500-1:1000)
Alexa Fluor 647 goat anti-mouse IgG1 #A21240 (IF: 1:500-1:1000)
Alexa Fluor 488 goat anti-mouse IgG2a #A21131 (IF: 1:500-1:1000)
Alexa Fluor 568 goat anti-mouse IgG1 #A21124 (IF: 1:500-1:1000)
Alexa Fluor 647 goat anti-rabbit IgG (H+L) #A21244 (IF: 1:500-1:1000)
Goat a-mouse IgG-HRP #HAF007 (IF: 1:10000)
Goat a-rabbit IgG-HRP #HAF008 (IF: 1:10000)

Validation

All antibodies used were validated by western blot by the commercial supplier as well as by our laboratory. For all antibodies used, molecular weight bands by western blot matched these expected from the theoretical MW of the corresponding proteins. Staining patterns in cells matched the expected and/or published patterns for all antibodies.

Eukaryotic cell lines

Policy information about cell lines and Sex and Gender in Research

Cell line source(s)	HeLa and U2OS cell lines were originally from ATCC (#CCL-2 and HTB-96, respectively).
Authentication	Cell lines were not authenticated.
Mycoplasma contamination	All cell lines were negative for mycoplasma contamination as determined by DAPI staining of DNA.
Commonly misidentified lines (See ICLAC register)	No commonly misidentified lines were used.
