# Supplementary material for: BICD2 phosphorylation regulates dynein function and centrosome separation in G2 and M
Source: Nat Commun. 2023 Apr 27;14:2434. doi: 10.1038/s41467-023-38116-1 (PMC10140047; doi:10.1038/s41467-023-38116-1)

Source data FIG. 1

FIG. 1A

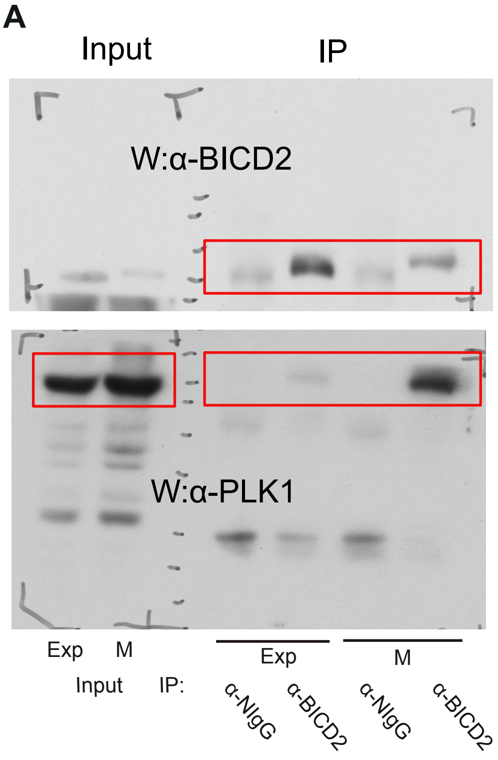

n=2

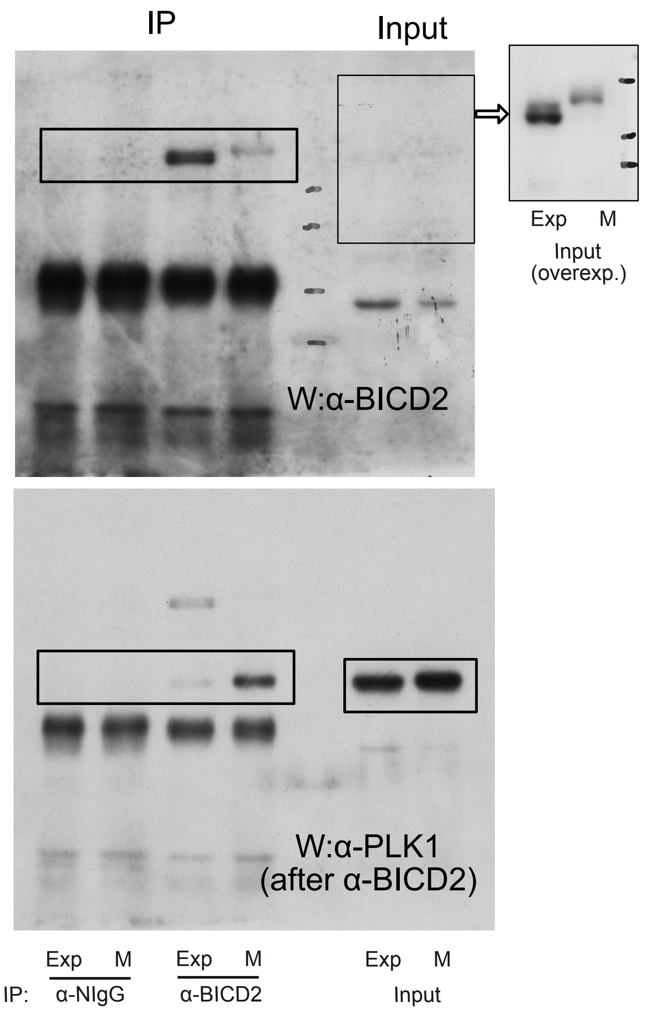

FIG. 1B

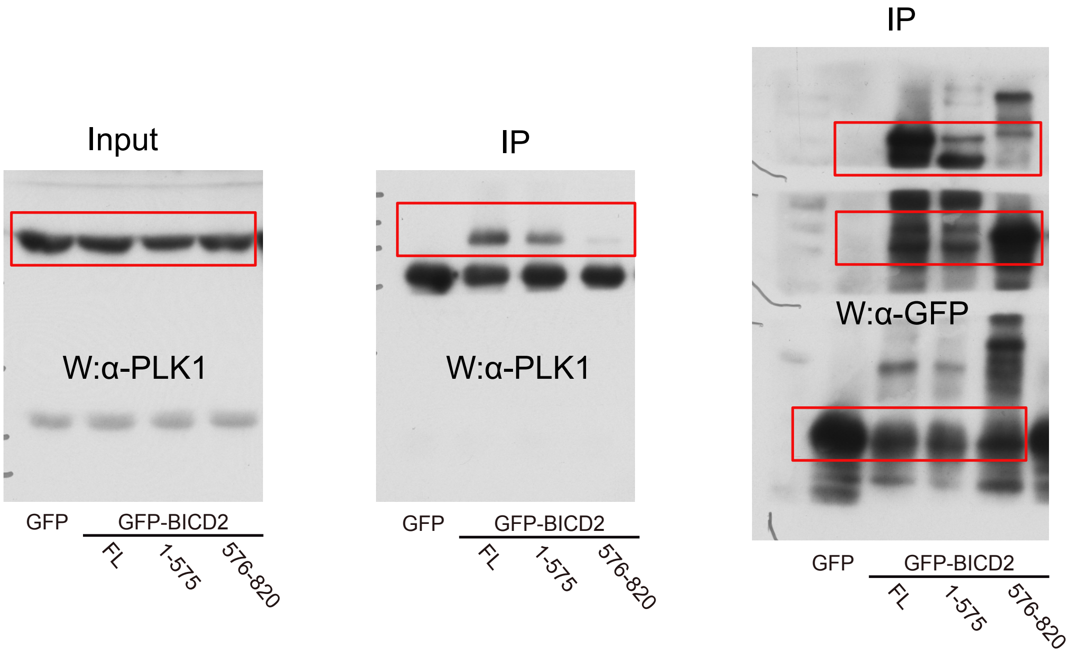

n=2

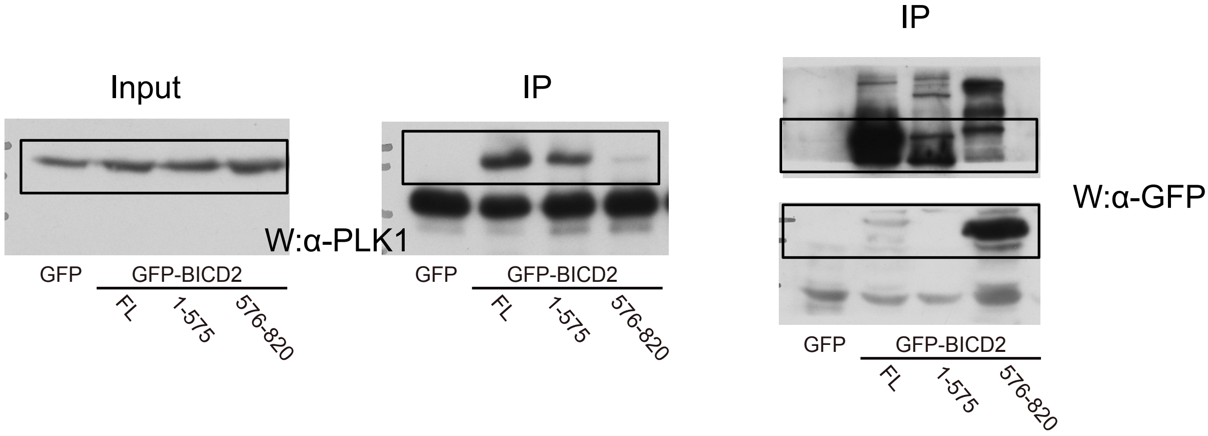

Source data FIG. 1

FIG. 1C

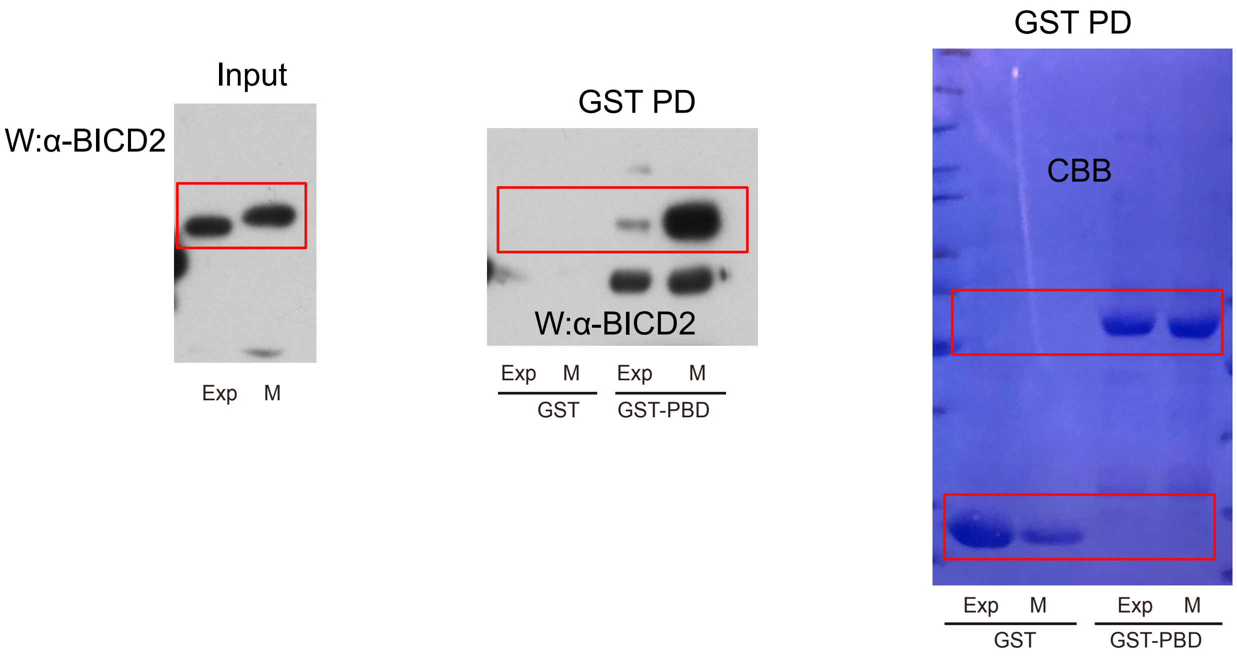

n=2

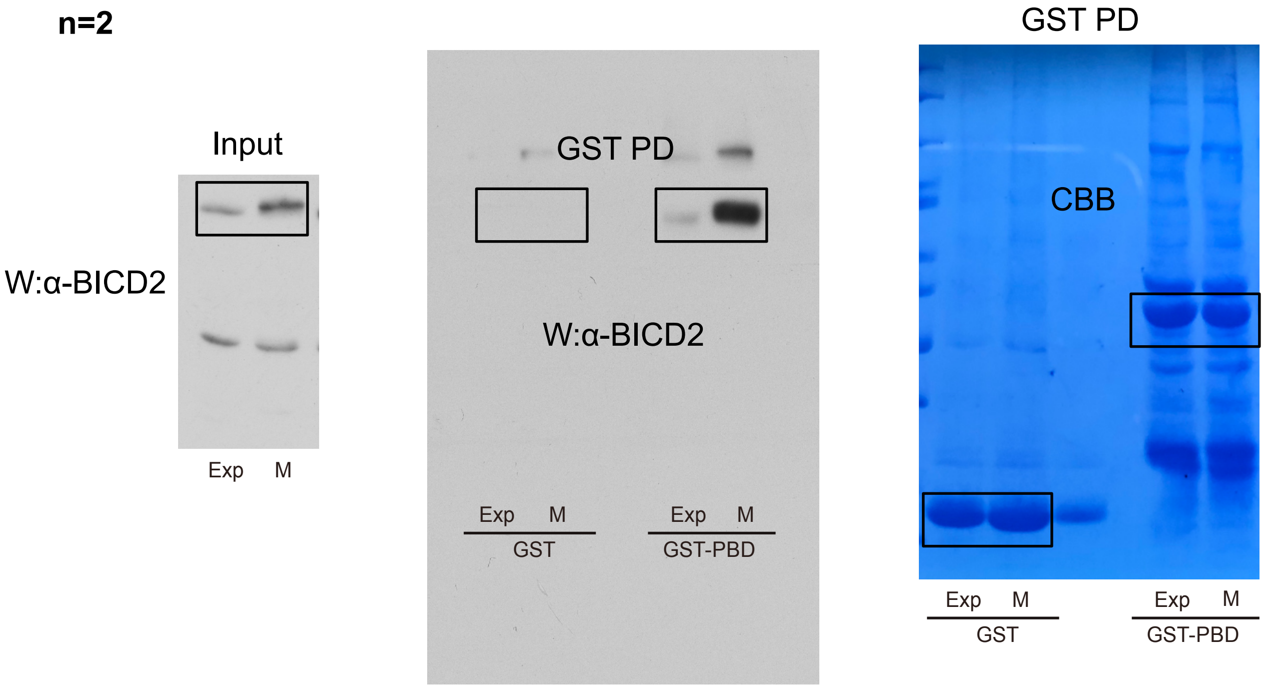

Source data FIG. 2

FIG. 2D

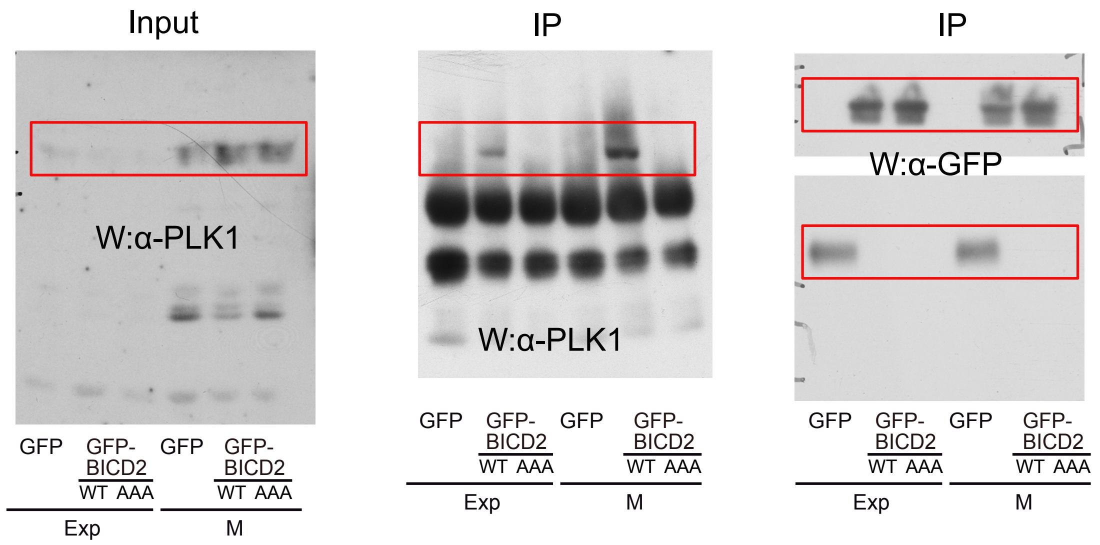

n=2

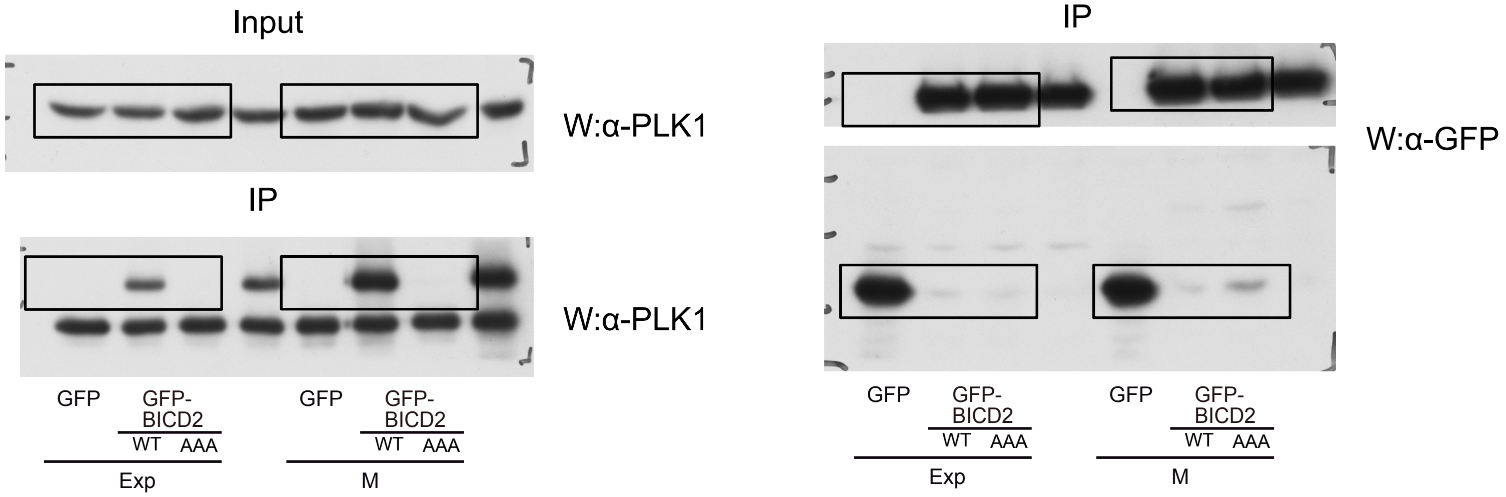

Source data FIG. 2

FIG. 2E

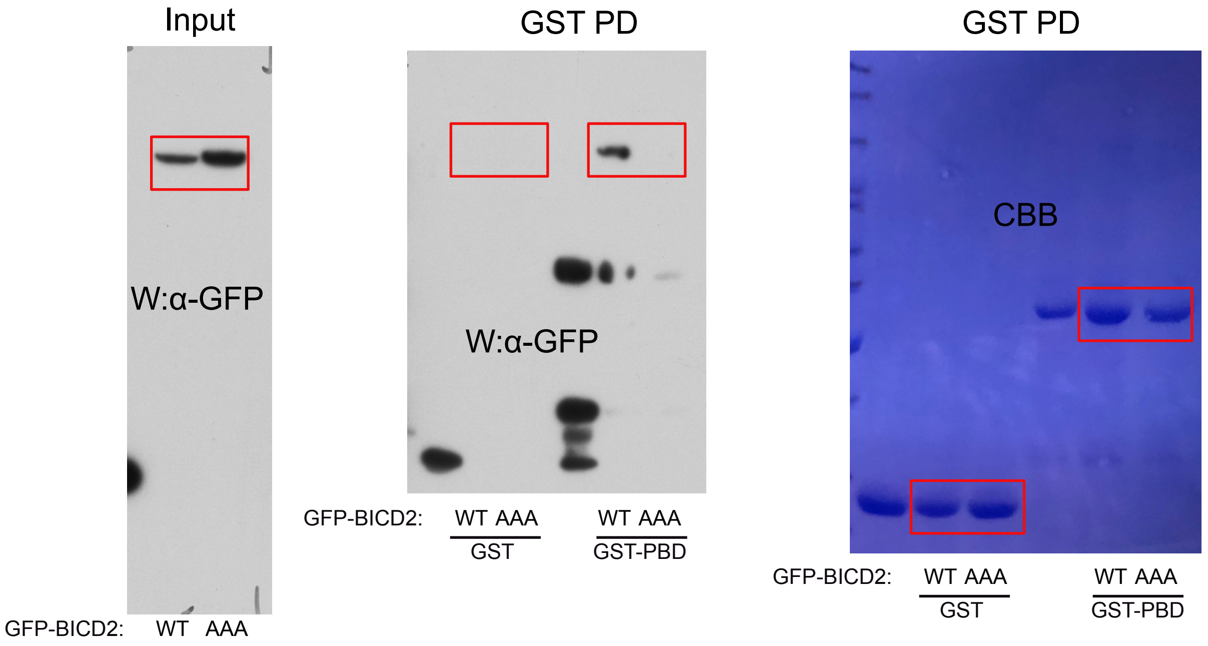

n=2

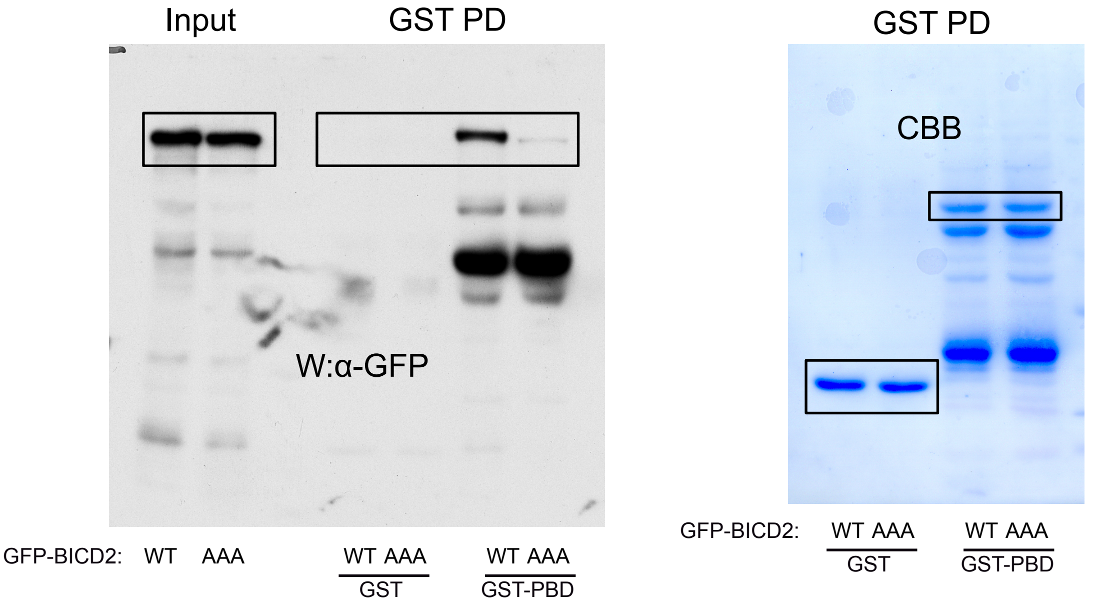

Source data FIG. 3

FIG. 3A

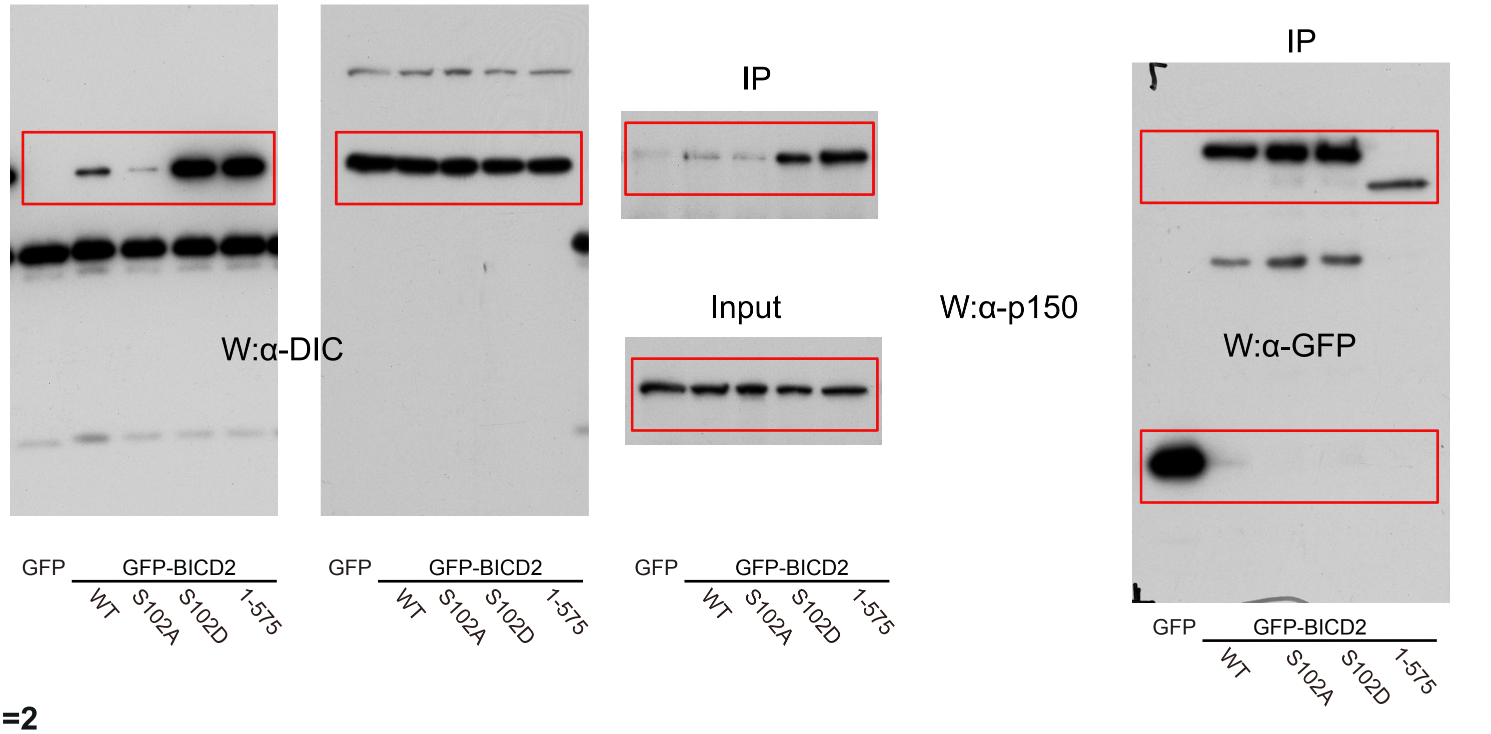

n=2

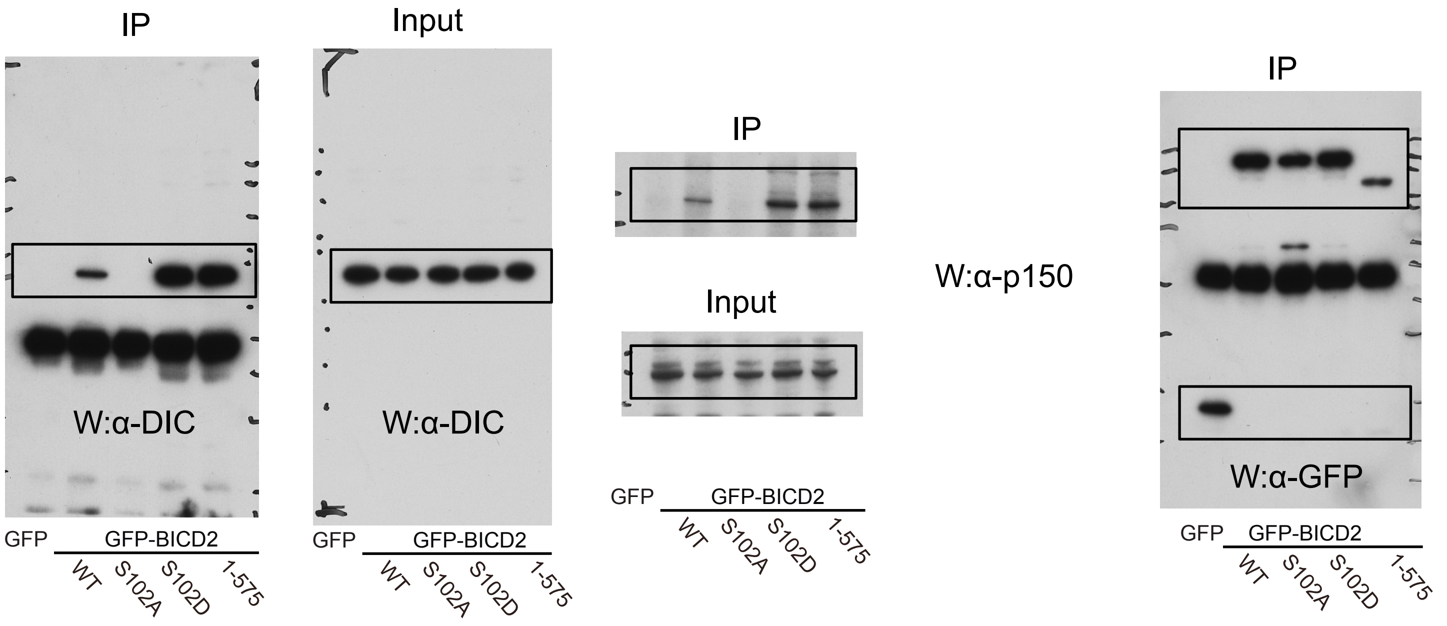

n=3

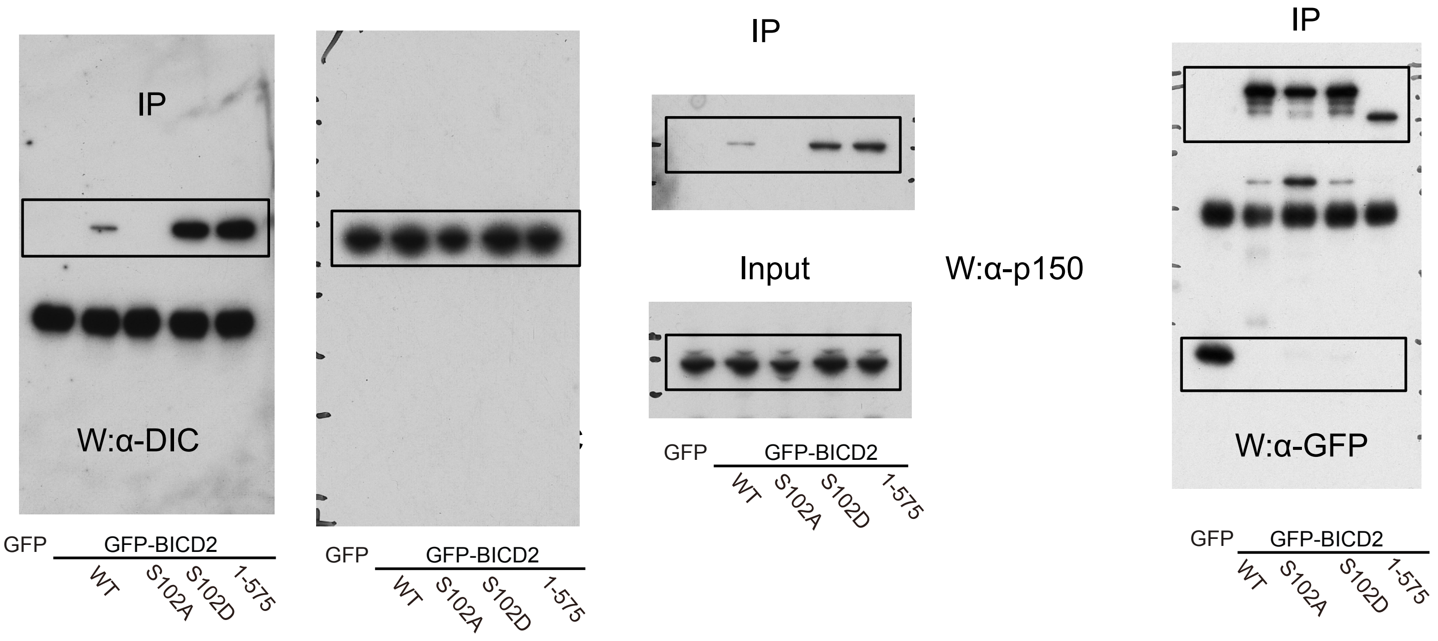

Source data FIG. 3

FIG. 3B

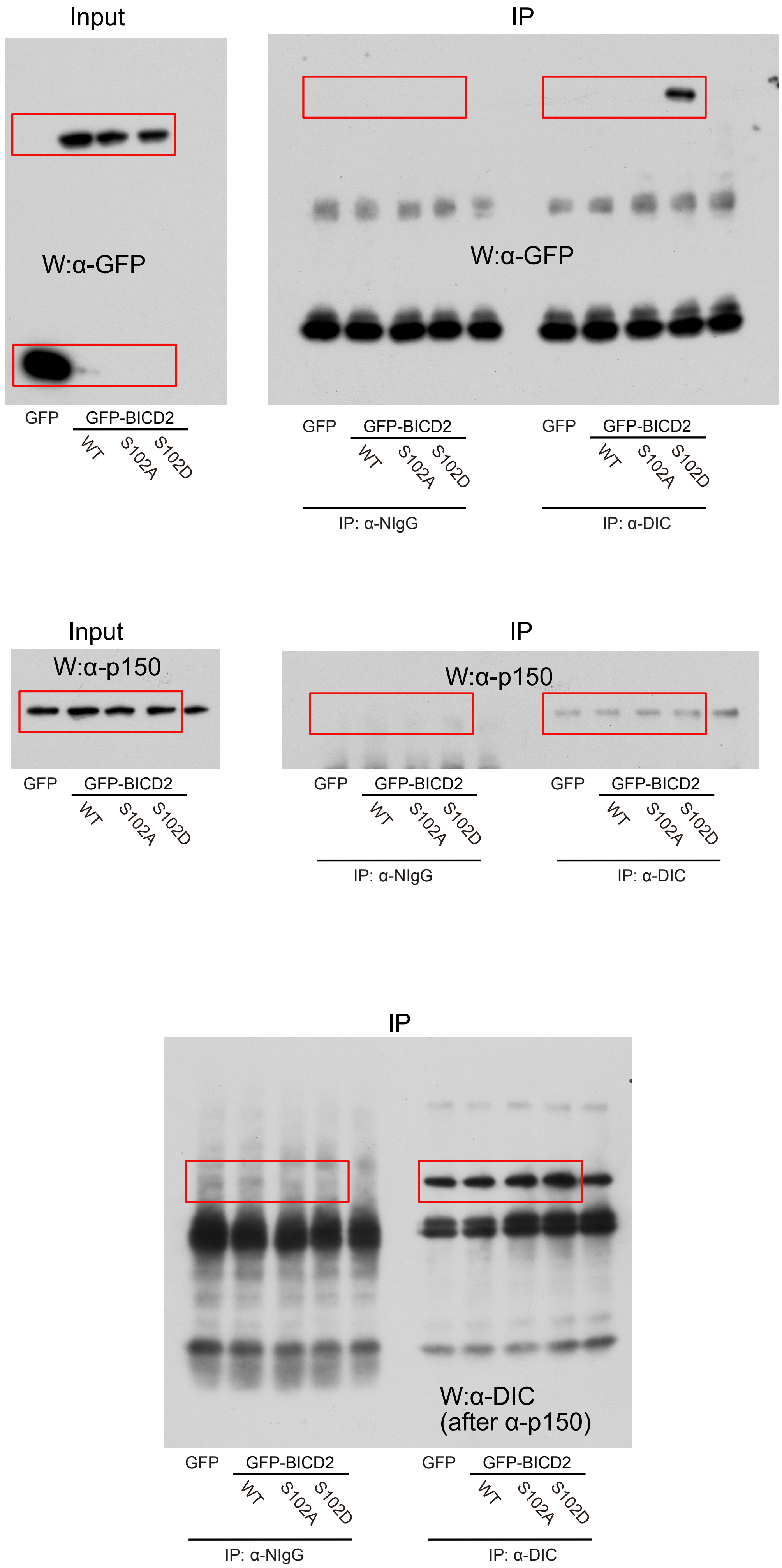

Source data FIG. 3

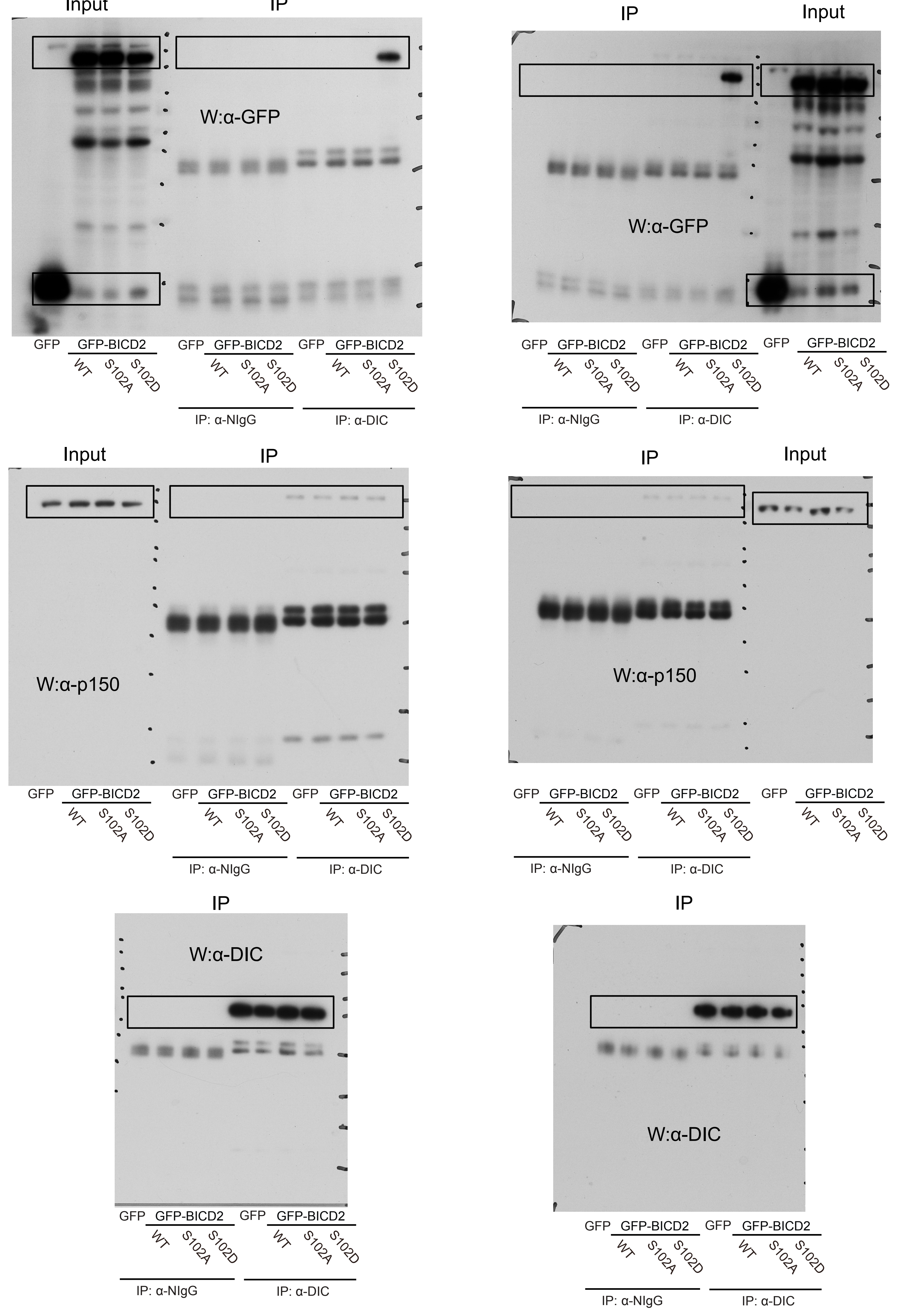

FIG. 3E

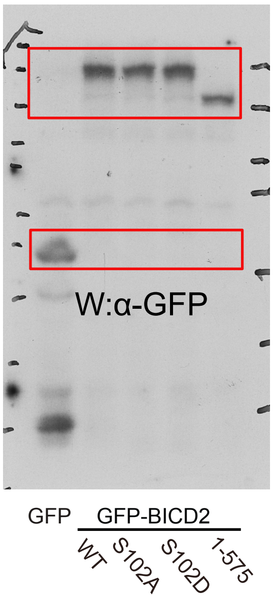

FIG. 4C

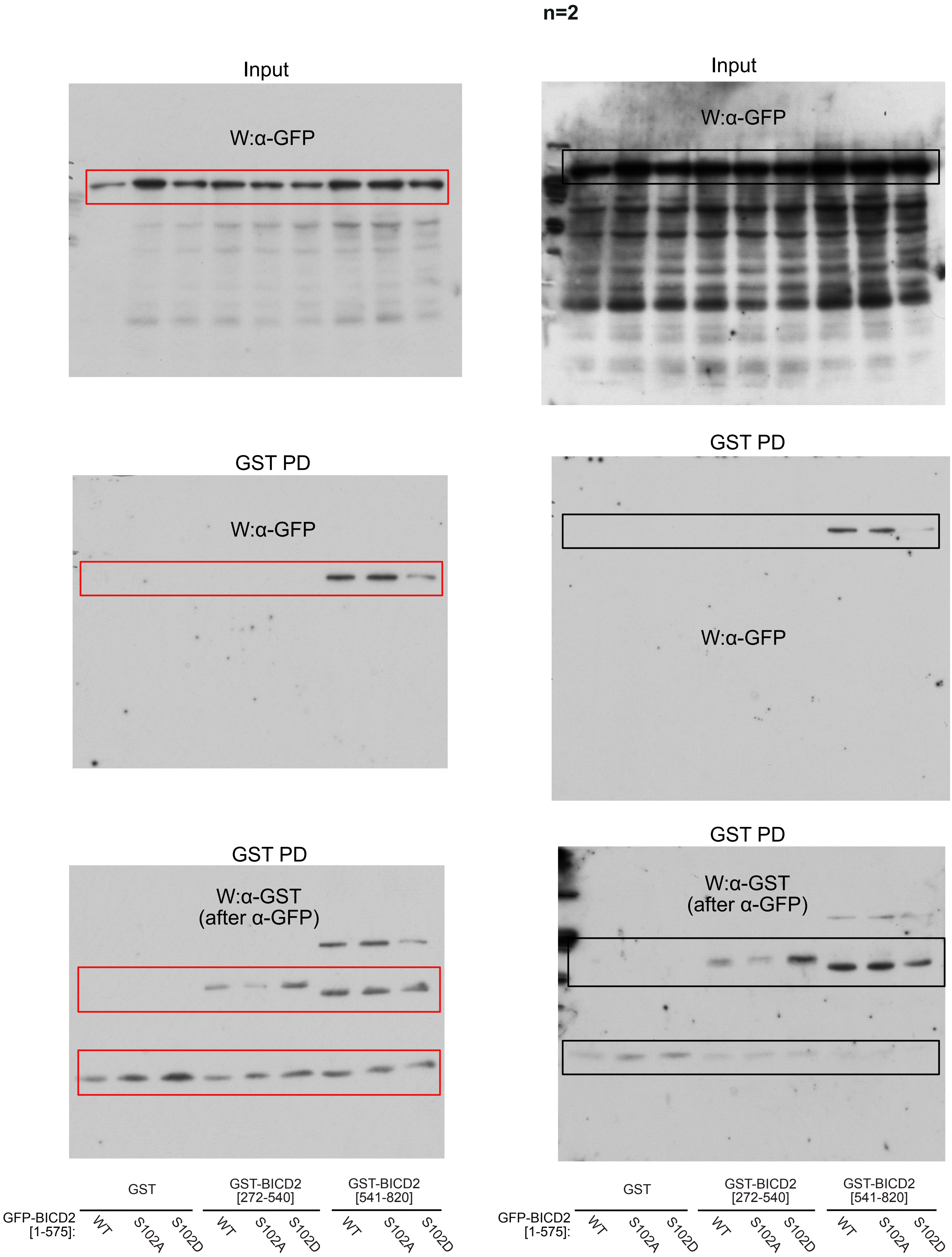
n=2
Input
W:α-GFP
Input
W:α-GFP
GST PD
W:α-GFP
GST PD
W:α-GFP
GST PD
W:α-GST
(after α-GFP)
GST PD
W:α-GST
(after α-GFP)
GST
GST-BICD2
[272-540]
GST-BICD2
[541-820]
GFP-BICD2
[1-575]:
WT
S102A
S102D
WT
S102A
S102D
WT
S102A
S102D
GST
GST-BICD2
[272-540]
GST-BICD2
[541-820]
GFP-BICD2
[1-575]:
WT
S102A
S102D
WT
S102A
S102D
WT
S102A
S102D

Source data FIG. 4

FIG. 4C

n=3

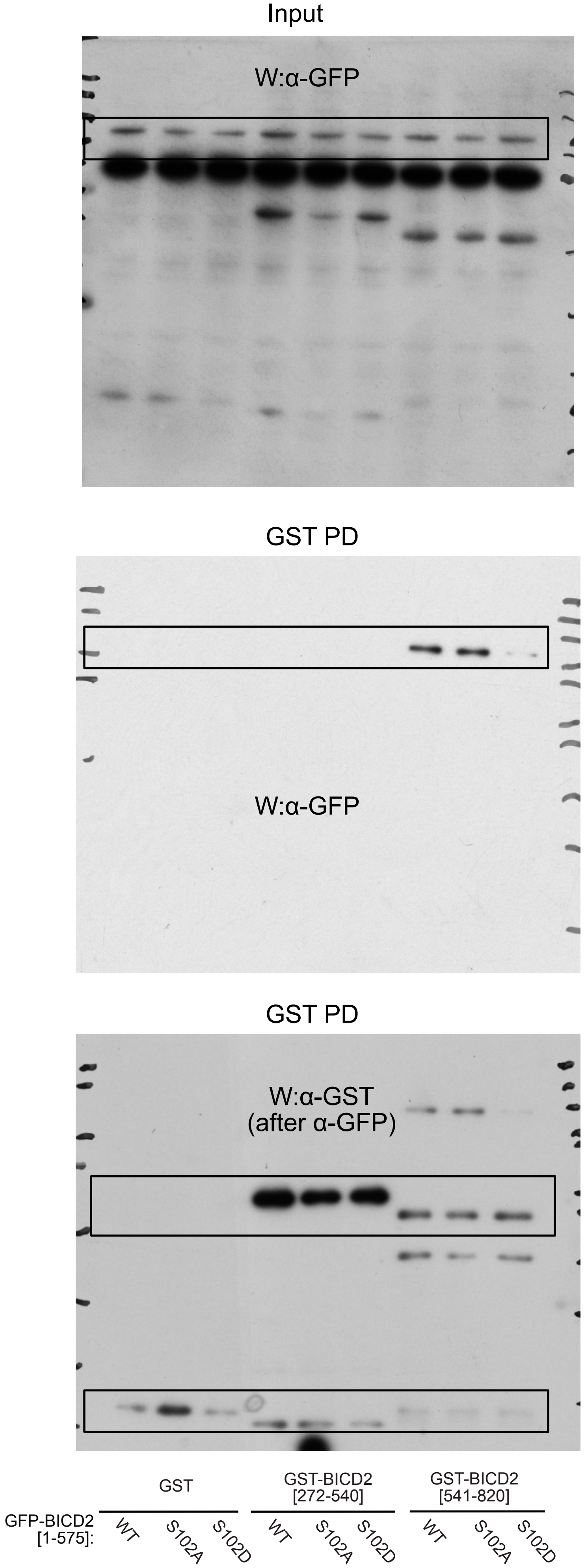

FIG. 5A

Source data FIG. 5

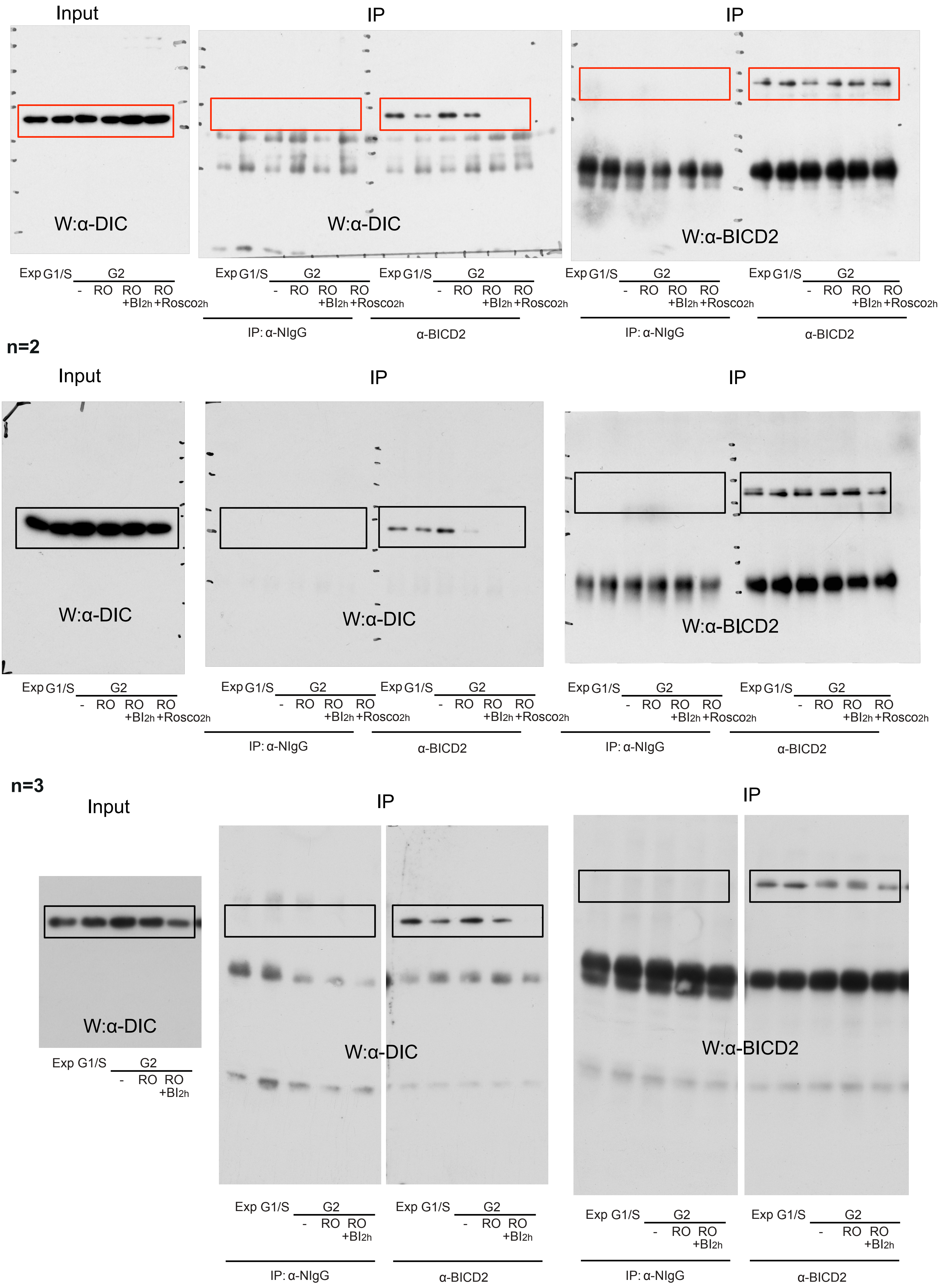

Source data FIG. 5

FIG. 5B

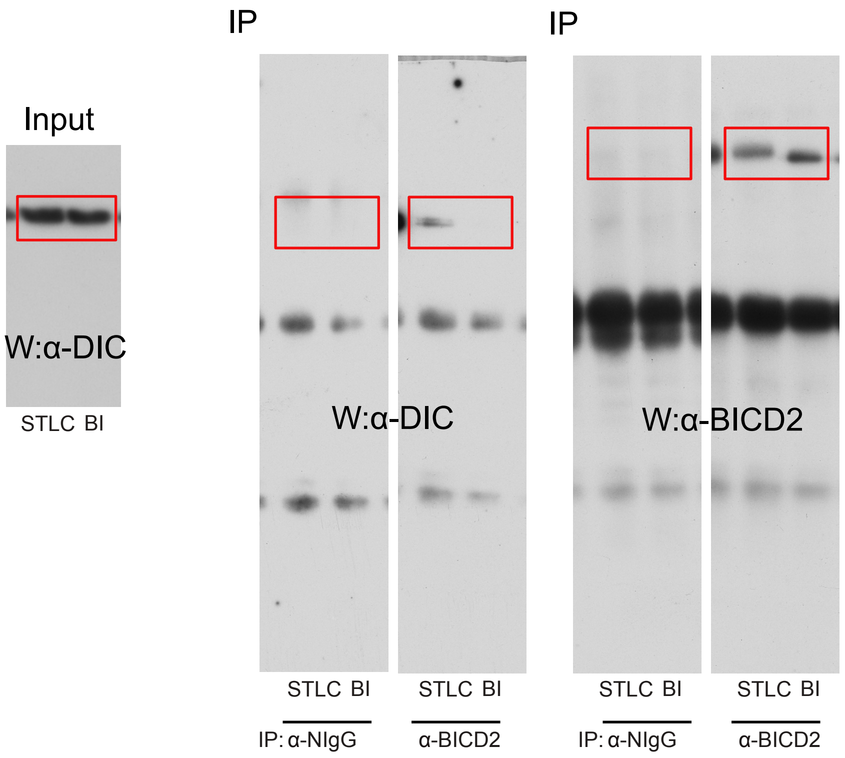

n=2

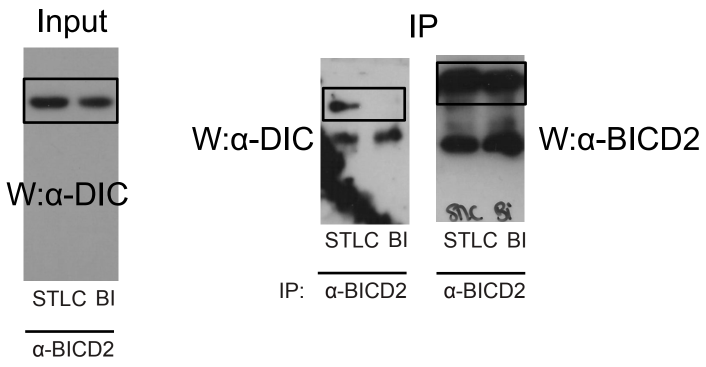

n=3

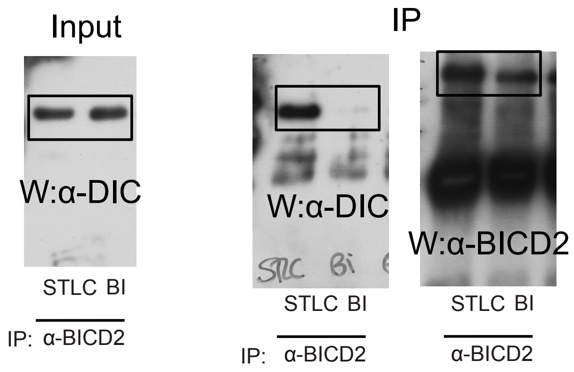

Source data FIG. 8

FIG. 8A

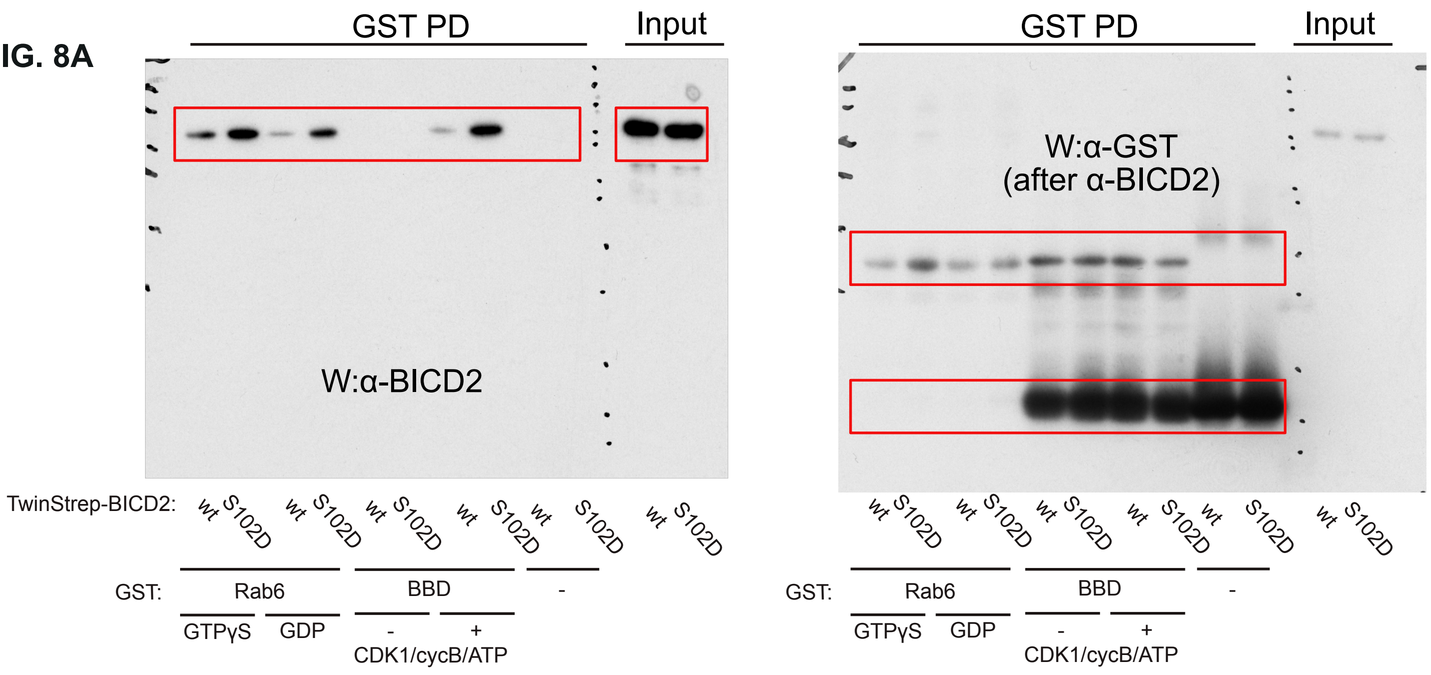

n=2

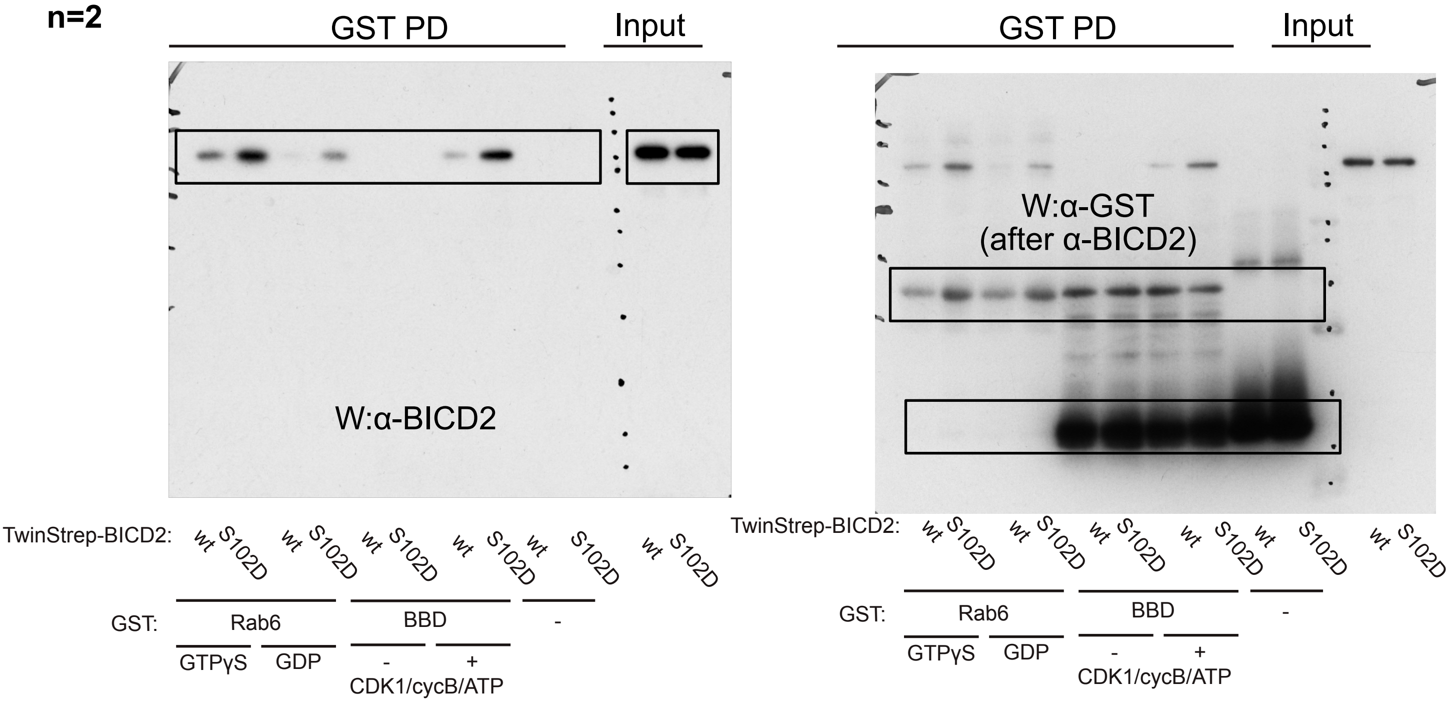

n=3

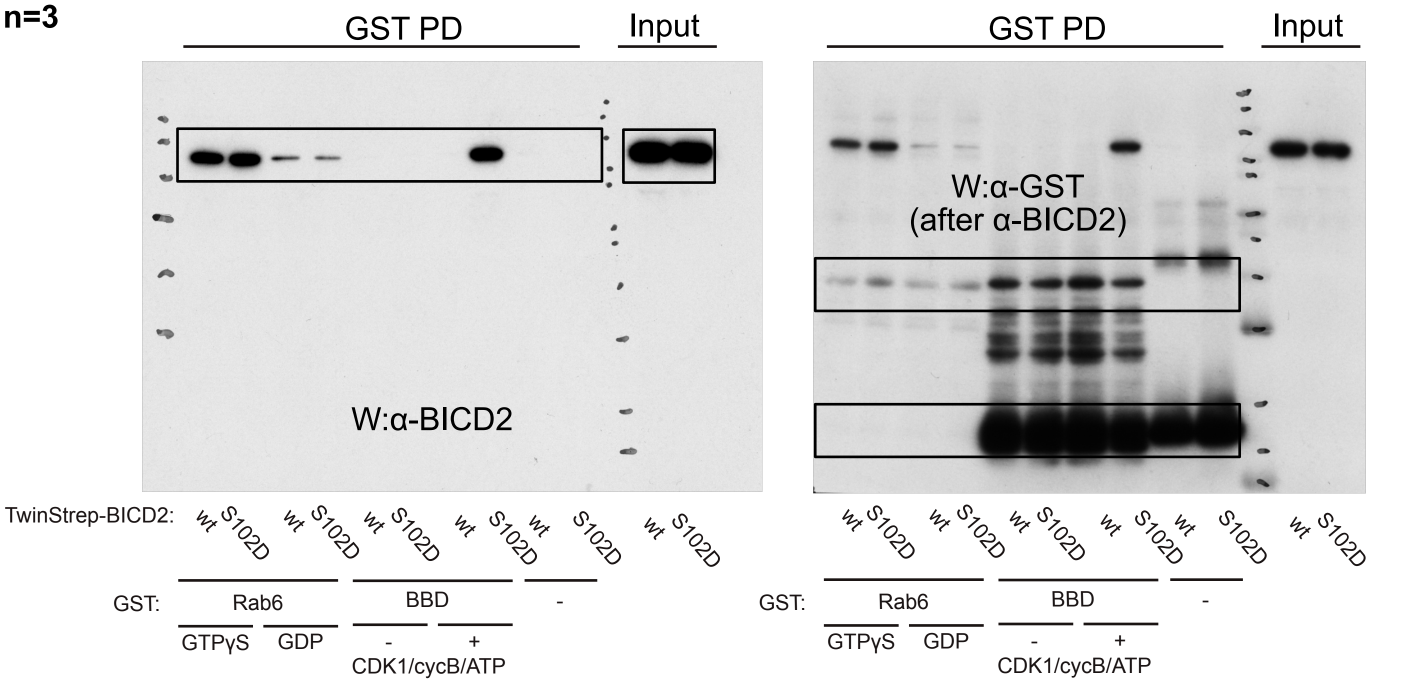

Supplement: Supplementary file 4 — Source Data [file 41467_2023_38116_MOESM4_ESM.zip › Source_data_1_Gallisa_etal.pdf]
