# Supplementary material for: BICD2 phosphorylation regulates dynein function and centrosome separation in G2 and M
Source: Nat Commun. 2023 Apr 27;14:2434. doi: 10.1038/s41467-023-38116-1 (PMC10140047; doi:10.1038/s41467-023-38116-1)
Supplement: Supplementary file 4 — Source Data [file 41467_2023_38116_MOESM4_ESM.zip › Source_data_2_Gallisa_etal.pdf]

FIGURE 1A

		Area (px)	Raw Integrated Density			
			Signal	-Control	Normalized	
**n=1 (FIG)**	**PLK1**					PLK1/BICD2
	Control Exp	18315	1215554			
	Exp	18315	1343509	127955	1	
	Control M	18315	1209265			
	M	18315	2949316	1740051	13.5989293	
	**BICD2**					
	Control Exp	15456	970817			
	Exp	15456	1882060	911243	1	1
	Control M	15456	924433			
	M	15456	1323372	398939	0.4377965	31.0622154
**n=2**	**PLK1**					PLK1/BICD2
	Control Exp	3034	95483669			
	Exp	3034	107755316	12271647	1	
	Control M	3034	96613561			
	M	3034	160549473	63935912	5.21005143	
	**BICD2**					
	Control Exp	2656	40456088			
	Exp	2656	121610492	81154404	1	1
	Control M	2656	42072589			
	M	2656	58379318	16306729	0.20093462	25.9290885

FIGURE 1C

		Area (px)	Raw Integrated Density			
			Signal	-Control	Normalized	
**n=1 (FIG)**		**BICD2**				BICD2/GST-PBD
	Control Exp	19656	1216865			
	Exp	19656	1445255	228390	1	
	Control M	19656	1202757			
	M	19656	3393042	2190285	9.59010902	
		**GST-PBD**				
	Control Exp	16926	2681964			
	Exp	16926	3223774	541810	1	1
	Control M	16926	2583400			
	M	16926	3293715	710315	1.31100386	7.31508833
**n=2**		**BICD2**				BICD2/GST-PBD
	Control Exp	12848	738710			
	Exp	12848	843084	104374	1	
	Control M	12848	742123			
	M	12848	1846936	1104813	10.5851361	
		**GST-PBD**				
	Control Exp	1824	219936			
	Exp	1824	335894	334070	1	1
	Control M	1824	225828			
	M	1824	323420	321596	0.96266052	10.9957102

FIGURE 2D

		Area (px)	Raw Integrated Density Signal	-Control	Normalized	PLK1/GFP-BICD2
**n=1 (FIG)**	**PLK1**					
	GFP Exp	1425	25006818			
	GFP-BICD2 wt Exp	1425	33231165	8224347	1	1
	GFP-BICD2 AAA Exp	1425	17702039	0	0	0
	GFP M	1425	29544752			
	GFP-BICD2 wt M	1425	57254642	27709890	3.36925108	3.36442824
	GFP-BICD2 AAA M	1425	18272888	0	0	0
	**GFP-BICD2**					
	GFP Exp	4896	244982			
	GFP-BICD2 wt Exp	4896	503793	258811	1	
	GFP-BICD2 AAA Exp	4896	528560	283578	1.09569531	
	GFP M	4896	242030			
	GFP-BICD2 wt M	4896	501212	259182	1.00143348	
	GFP-BICD2 AAA M	4896	521466	279436	1.07969136	
**n=2**	**PLK1**					PLK1/GFP-BICD2
	GFP Exp	28566	1028593			
	GFP-BICD2 wt Exp	28566	1974099	945506	1	1
	GFP-BICD2 AAA Exp	28566	1051498	22905	0.02422512	0.02238228
	GFP M	28566	1139839			
	GFP-BICD2 wt M	28566	3749699	2609860	2.76027862	2.58009304
	GFP-BICD2 AAA M	28566	1215258	75419	0.07976576	0.08230487
	**GFP-BICD2**					
	GFP Exp	24064	1009371			
	GFP-BICD2 wt Exp	24064	4099115	3089744	1	
	GFP-BICD2 AAA Exp	24064	4353509	3344138	1.08233498	
	GFP M	24064	1402507			
	GFP-BICD2 wt M	24064	4708029	3305522	1.06983685	
	GFP-BICD2 AAA M	24064	4396932	2994425	0.96914987	

FIGURE 2E

		Area (px)	Raw Integrated Density			
			Signal	-Control	Normalized	
**n=1 (FIG)**		**GFP-BICD2**				GFP-BICD2/GST-PBD
	Control GFP-BICD2 wt	7560	465593			
	Control GFP-BICD2 AAA	7560	457047			
	GFP-BICD2 wt	7560	994681	529088	1	1
	GFP-BICD2 AAA	7560	459827	2780	0.00525432	0.00720567
		**GST-PBD**				
	Control GFP-BICD2 wt	17856	2958520			
	Control GFP-BICD2 AAA	17856	3006806			
	GFP-BICD2 wt	17856	3495297	536777	1	
	GFP-BICD2 AAA	17856	3398220	391414	0.72919294	
**n=2**		**GFP-BICD2**				GFP-BICD2/GST-PBD
	Control GFP-BICD2 wt	3312	25650146			
	Control GFP-BICD2 AAA	3312	26334973			
	GFP-BICD2 wt	3312	102377529	76727383	1	1
	GFP-BICD2 AAA	3312	28914810	2579837	0.03362342	0.0355524
		**GST-PBD**				
	Control GFP-BICD2 wt	2604	93818			
	Control GFP-BICD2 AAA	2604	114090			
	GFP-BICD2 wt	2604	314046	220228	1	
	GFP-BICD2 AAA	2604	322369	208279	0.94574259	

FIGURE 3C	Area (px)	Raw Integrated Density					
		Signal	Background	S-BCK	Normalized	DIC/GFP	Normalized
**n=1 (FIG)**							
**DIC**							
wt	3120	327374	188739	138635	1	0.33235044	1
S102A	3120	242549	192046	50503	0.36428752	0.11085673	0.33355373
S102D	3120	658570	211817	446753	3.22251235	0.95263409	2.86635426
1-575	3120	673677	199575	474102	3.41978577	2.02313732	6.08736168
**GFP**							
wt	3502	656610	239475	417135	1		
S102A	3652	701251	245681	455570	1.09214043		
S102D	3652	706726	237760	468966	1.12425474		
1-575	3502	458597	224257	234340	0.56178455		
**n=2**							
**DIC**							
wt	3752	95620647	57877879	37742768	1	0.5048586	1
S102A	3752	61430860	56758110	4672750	0.12380518	0.07774677	0.15399712
S102D	3752	168604370	58322707	110281663	2.92192833	1.48837614	2.94810495
1-575	3752	175083974	59625875	115458099	3.05907874	4.61654271	9.14422912
**GFP**							
wt	2982	121986005	47226918	74759087	1		
S102A	2982	107132869	47030695	60102174	0.80394473		
S102D	2982	121444822	47349531	74095291	0.99112087		
1-575	2982	71836815	46827169	25009646	0.33453654		
n=3							
**DIC**							
wt	2640	57686662	43600655	14086007	1	0.21587827	1
S102A	2640	45455499	43486817	1968682	0.13976154	0.03350465	0.15520159
S102D	2640	117604152	46284967	71319185	5.06312293	1.13854463	5.27401231
1-575	2640	129864179	49774917	80089262	5.68573209	2.0777303	9.62454596
**GFP**							
wt	2376	77501990	12252221	65249769	1		
S102A	2376	71254055	12495585	58758470	0.90051614		
S102D	2376	75024501	12383843	62640658	0.96001348		
1-575	2376	50317502	11770987	38546515	0.59075328		

FIGURE 3D	Area (px)	Raw Integrated Density					
		Signal	Background	S-BCK	Normalized	GFP/DIC	Normalized
**n=1 (FIG)**							
**GFP**							
wt	2736	162480	159118	3362	1	0.01039496	1
S102A	2736	161718	158673	3045	0.90571089	0.00817342	0.78628704
S102D	2736	396824	159848	236976	70.4866151	0.5144785	49.4930766
**DIC**							
wt	3280	530504	207078	323426	1		
S102A	3280	578543	205994	372549	1.15188327		
S102D	3280	672547	211933	460614	1.42417122		
**n=2**							
**GFP**							
wt	2040	25542176	24245458	1296718	1	0.01336403	1
S102A	2040	26721176	24115797	2605379	2.00921018	0.02488914	1.86239806
S102D	2040	81058457	24020248	57038209	43.9865946	0.56411646	42.2115618
**DIC**							
wt	2970	146459691	49429209	97030482	1		
S102A	2970	153558468	48879111	104679357	1.07882961		
S102D	2970	149106763	47996065	101110698	1.04205087		
**n=3**							
**GFP**							
wt	2356	29504482	28115147	1389335	1	0.0164223	1
S102A	2356	30723490	27843296	2880194	2.07307381	0.03549784	2.16156386
S102D	2356	86631274	27742466	58888808	42.3863273	0.92558309	56.3613725
**DIC**							
wt	2666	127076154	42475616	84600538	1		
S102A	2666	123859946	42722783	81137163	0.95906202		
S102D	2666	105380151	41756681	63623470	0.75204569		

FIGURE 3E	GFP-MTD			GFP-MTD-BICD2 wt			GFP-MTD-BICD2 S102A			GFP-MTD-BICD2 S102D			GFP-MTD-BICD2 1-575		
	n=1	n=2	n=3	n=1	n=2	n=3	n=1	n=2	n=3	n=1	n=2	n=3	n=1	n=2	n=3
Clustered	9	7	7	81	68	72	68	55	56	60	71	96	121	98	131
Unclustered	106	98	100	70	79	69	81	75	78	45	49	45	24	18	19
TOTAL	115	105	107	151	147	141	149	130	134	105	120	141	145	116	150
TOTAL 3 exps clustered			23			221			179			227			350
TOTAL 3 exps unclustered			304			218			234			139			61
%															
Clustered	7.8	6.7	6.5	53.6	46.3	51.1	45.6	42.3	41.8	57.1	59.2	68.1	83.4	84.5	87.3
Unclustered	92.2	93.3	93.5	46.4	53.7	48.9	54.4	57.7	58.2	42.9	40.8	31.9	16.6	15.5	12.7
TOTAL	100.0	100.0	100.0	100.0	100.0	100.0	100.0	100.0	100.0	100.0	100.0	100.0	100.0	100.0	100.0

FIGURE 4C	Area (px)	Raw Integrated Density					
		Signal	Background	S-BCK	Normalized	GFP BICD2 [1	Normalized
**n=1 (FIG)**							
**GFP-BICD2**							
GFp-BICD2 [1-575] wt	2296	75821253	38626110	37195143	1	1.21511437	1
GFp-BICD2 [1-575] S102A	2296	81879574	38777063	43102511	1.15882095	1.50807745	1.24109918
GFp-BICD2 [1-575] S102D	2296	52667336	38318343	14348993	0.38577599	0.56308634	0.46340193
GST-BICD2[541-820]	3096	83333334	52722929	30610405	1		
GST-BICD2[541-820]	3096	79990595	51409496	28581099	0.93370535		
GST-BICD2[541-820]	3096	76881007	51398249	25482758	0.8324868		
**n=2**							
**GFP-BICD2**							
GFp-BICD2 [1-575] wt	2236	224640	118949	105691	1	0.00239173	1
GFp-BICD2 [1-575] S102A	2236	209669	118428	91241	0.8632807	0.0021199	0.88634316
GFp-BICD2 [1-575] S102D	2236	128059	117257	10802	0.10220359	0.00040847	0.17078396
GST-BICD2[541-820]	3600	96416120	52225998	44190122	1		
GST-BICD2[541-820]	3600	97310480	54270176	43040304	0.9739802		
GST-BICD2[541-820]	3600	79463115	53018072	26445043	0.59843788		
**n=3**							
**GFP-BICD2**							
GFp-BICD2 [1-575] wt	1400	27030832	9120913	17909919	1	1.16565625	1
GFp-BICD2 [1-575] S102A	1400	27749063	9223324	18525739	1.0343843	1.71070291	1.46758781
GFp-BICD2 [1-575] S102D	1400	10837182	9205272	1631910	0.09111767	0.10613985	0.09105587
GST-BICD2[541-820]	1534	38294913	22930247	15364666	1		
GST-BICD2[541-820]	1534	33542109	22712795	10829314	0.70481936		
GST-BICD2[541-820]	1534	37863010	22487917	15375093	1.00067863		

FIGURE 5A	Area (px)	Raw Integrated Density				
		Signal	Background	S-BCK	Normalized	DIC/BICD2
**n=1 (FIG)**						
**DIC**						
Exp	1708	167331	44496	122835	1	1
G1/S	1708	97601	43423	54178	0.44106321	0.35590669
G2	1708	177758	41017	136741	1.11320878	1.53338125
G2 (RO)	1708	116056	40360	75696	0.6162413	0.55388569
G2 (RO) + BI	1708	39924	39312	612	0.00498229	0.00416786
G2 (RO) + Rosco	1708	39424	39156	268	0.00218179	0.00158774
**BICD2**						
Exp	1740	134790	44000	90790	1	
G1/S	1740	154679	42166	112513	1.23926644	
G2	1740	108946	43034	65912	0.72598304	
G2 (RO)	1740	142592	41581	101011	1.11257848	
G2 (RO) + BI	1740	150801	42270	108531	1.19540698	
G2 (RO) + Rosco	1740	171243	46484	124759	1.37414914	
**n=2**						
**DIC**	1608	142830	73354	69476	1	1
G1/S	1608	130246	74431	55815	0.80337095	0.73340792
G2	1608	175773	75471	100302	1.44369279	1.18544073
G2 (RO)	1608	82033	74477	7556	0.10875698	0.09762094
G2 (RO) + BI	1608	72363	71654	709	0.01020496	0.00797664
G2 (RO) + Rosco	1608	71794	71530	264	0.00379987	0.00449319
**BICD2**						
Exp	1624	171438	52647	118791	1	
G1/S	1624	184356	54233	130123	1.09539443	
G2	1624	202506	57836	144670	1.2178532	
G2 (RO)	1624	188961	56619	132342	1.1140743	
G2 (RO) + BI	1624	209243	57267	151976	1.27935618	
G2 (RO) + Rosco	1624	156464	56003	100461	0.84569538	
**n=3**						
**DIC**						
Exp	2016	247770	109657	138113	1	1
G1/S	2016	189621	108241	81380	0.58922766	0.53825819
G2	2016	256758	105051	151707	1.09842665	1.18861996
G2 (RO)	2016	174836	104613	70223	0.50844598	0.52189863
G2 (RO) + BI	2016	104047	102703	1344	0.00973116	0.01591324
**BICD2**						
Exp	2880	282540	160335	122205	1	
G1/S	2880	292263	158486	133777	1.09469334	
G2	2880	270908	157976	112932	0.92411931	
G2 (RO)	2880	277711	158656	119055	0.97422364	
G2 (RO) + BI	2880	229524	154794	74730	0.61151344	

FIGURE 5B	Area (px)	Raw Integrated Density				
		Signal	Background	S-BCK	Normalized	DIC/BICD2
**n=1 (FIG)**						
**DIC**						
STLC	2738	215169	140861	74308	1	1
BI	2738	140424	139526	898	0.01208484	0.01162245
**BICD2**						
STLC		300409	153187	147222	1	
BI		302774	149695	153079	1.03978346	
**n=2**						
**DIC**						
STLC	1652	255736	172292	83444	1	1
BI	1652	172660	171855	805	0.00964719	0.01043442
**BICD2**						
STLC	3036	674318	369369	304949	1	
BI	3036	658806	376864	281942	0.9245546	
**n=3**						
**DIC**						
STLC	3872	631009	194359	436650	1	1
BI	3872	189609	174561	15048	0.03446238	0.03847023
**BICD2**						
STLC	3827	756679	707927	48752	1	
BI	3827	675979	632306	43673	0.89581966	

FIGURE 6B

	Negative			Weak/partial			High		
	n=1	n=2	n=3	n=1	n=2	n=3	n=1	n=2	n=3
**BICD2**									
DMSO	0	0	0	20	40	30	80	60	70
Roscovitine	100	90	90	0	10	10	0	0	0
RO-3306	20	40	40	70	60	40	10	0	20
BI2536	50	70	50	50	30	40	0	0	10
**DIC**									
DMSO	0	10	10	50	50	50	50	40	40
Roscovitine	100	90	90	0	10	10	0	0	0
RO-3306	20	60	40	60	40	40	20	0	20
BI2536	70	80	40	30	20	50	0	0	10

FIGURE 6E and 6F	Negative			Weak/partial			High		
	n=1	n=2	n=3	n=1	n=2	n=3	n=1	n=2	n=3
**GFP**									
Ci GFP-O	100	100	100	0	0	0	0	0	0
BICD2i GFP-O	100	100	100	0	0	0	0	0	0
BICD2i GFP-BICD2-WT	0	7.1	0	20	7.1	10	80	85.8	90
BICD2i GFP-BICD2-AAA	70	73.3	72.7	30	26.7	27.3	0	0	0
BICD2i GFP-BICD2-102A	60	63.6	70	40	36.4	30	0	0	0
BICD2i GFP-BICD2-102D	9	0	0	36.4	33.3	10	54.6	66.7	90
**DIC**									
Ci GFP-O	25	0	0	37.5	40	50	37.5	60	50
BICD2i GFP-O	40	30	80	40	40	20	20	30	0
BICD2i GFP-BICD2-WT	0	7.1	0	40	35.7	40	60	57.2	60
BICD2i GFP-BICD2-AAA	60	73.3	54.5	40	26.7	45.5	0	0	0
BICD2i GFP-BICD2-102A	70	27.3	60	30	72.7	40	0	0	0
BICD2i GFP-BICD2-102D	9.1	8.3	0	27.3	33.3	40	63.6	58.4	60

FIGURE 7B

	Distance centrosome-NE in G2									
	Ci GFP-O	Ci GFP-WT	Ci GFP-AAA	Ci GFP-A	Ci GFP-D	BICD2i GFP-C	ICD2i GFP-W	ICD2i GFP-AA	BICD2i GFP-A	BICD2i GFP-D
n=1	1.295	0.432	0.1	0	0	2.608	0	5.503	3.825	0
	0.863	0.41	0.984	0	0.546	2.947	0	6.452	4.659	0
	3.808	0	3.006	1.351	0.546	6.757	0	5.225	0	0.819
	4.472	0	3.496	1.295	0.305	6.622	0	4.507	0	0.715
	0.965	0	0	1.101	0	1.901	0	5.198	1.229	0
	0.819	0.965	0	0.863	0	2.321	0	4.411	1.775	0
	5.532	0	3.276	1.644	0.546	4.043	1.101	0.796	0	0.41
	5.963	0	2.472	1.954	0.579	4.826	1.126	1.916	0	0.65
	6.694	0.683	11.137	0.819	0	20.042	0	0	2.016	0
	6.059	1.526	13.119	1.229	0	19.635	0	0	2.321	0
	2.325	0	3.413	2.016	0	6.565	0	3.377	11.975	0
	2.205	0	2.626	1.775	0	6.565	0	2.918	13.981	0
	0	0	1.775	3.358	0	4.152	0	2.066	4.893	0.772
	0	0	4.31	3.48	0	3.573	0	2.947	5.543	0.683
	0.563	0	0.546	2.744	0	1.229	0	3.98	1.229	0
	0.83	0	0.305	3.302	0	1.126	0	3.413	1.78	0
	2.325	0.137	0	1.351	0.492	13.874	0	1.821	2.333	0
	2.87	0.432	0	1.71	0.495	13.741	0	2.251	1.451	0
	0	0	2.472	3.302	0	1.221	1.47	1.661	2.124	1.821
	0	0	1.508	3.488	0.273	1.01	1.451	4.338	1.556	2.184
n=2	4.402	0	2.148	1.365	5.761	5.331	0	0	0	2.06
	4.387	0	1.556	1.721	5.312	6.082	0	0	1.06	0.683
	2.205	0	0	0	0	1.931	1.78	0	6.374	0
	3.052	0	0	0	0	1.78	2.048	0	5.189	0
	1.666	1.158	1.236	2.902	0	3.844	0	2.205	0.819	1.727
	1.666	1.451	1.295	3.391	1.477	3.413	0	2.388	1.295	1.689
	2.744	0.984	2.51	0	1.556	4.776	0	0	0	3.437
	1.916	0.193	2.626	0	1.092	5.436	0	0.546	0	4.072
	4.768	0	1.78	2.048	0	7.046	1.174	3.583	0	0.735
	4.893	0	0.432	1.649	0	6.382	0.683	5.153	0	0.735
	0.61	1.857	1.101	0	3.113	3.583	0	0	1.101	0.305
	1.04	1.101	1.101	0	2.59	3.198	0	0.819	1.236	0.579
	0	0	3.421	1.066	0	4.659	0	1.451	1.78	0
	0	0	6.485	1.477	0	4.463	0	2.55	1.236	0
	5.832	3.445	0.874	0.546	4.241	6.288	0	4.949	0.696	4.635
	8.927	2.902	0.994	2.918	3.861	6.903	0	4.519	1.166	5.275
	0.546	0	0.956	0	1.288	5.577	0	2.356	0.965	0
	0.965	0	0	0	1.837	5.628	0	1.526	0.579	0
	0.696	2.08	1.101	2.51	0	3.184	0	0	6.217	1.236
	0.696	2.73	1.066	1.737	0	2.94	0	0.796	6.062	1.229
n=3	0	0	0	0	0	0	0	2.754	3.132	0
	0	0	0	0	0	0	0	3.207	2.722	0
	0	0	1.376	0	1.031	2.409	0	0	0.497	0.362
	0	0	1.557	0	0.973	2.672	0	0	0.763	0.36
	0	0	0.615	1.283	0	0	1.283	1.473	1.964	0
	0	0	1.412	1.715	0	0	1.055	1.984	1.717	0
	0	1.109	1.453	0.573	0	1.376	0	0	1.197	0
	0	0.919	3.106	1.399	0	2.259	0	0	1.109	0
	0	0	3.592	1.376	0	0	0	0.954	1.785	0
	0	0	2.848	1.917	0	0	0	1.389	1.997	0
	0	0.546	2.427	1.589	0	1.185	0	1.468	1.838	0
	0	0.603	3.252	2.851	0	2.023	0	1.268	2.218	0
	0	0	0.704	0	0	3.129	0	0	1.112	0
	0	0	0.683	0	0	2.403	0	0	1.4	0
	0	0	1.603	1.846	0	2.428	0	2.035	0.787	0
	0	0	1.333	1.657	0	1.999	0	2.296	1.093	0
	0	0	1.908	0	0	1.538	0	2.591	1.122	0.256
	0	0	2.371	0	0	2.751	0	2.678	0	0.256
	0	0.519	0	0.853	0	0.54	0	1.112	1.657	0.675
	0	1.569	0	2.403	0	0.787	0	1.291	1.26	0.675

FIGURE 7C

	Distance centrosome-centrosome in G2									
	Ci GFP-O	Ci GFP-WT	Ci GFP-AAA	Ci GFP-A	Ci GFP-D	BICD2i GFP-O	ICD2i GFP-W	ICD2i GFP-AA	BICD2i GFP-A	BICD2i GFP-D
**n=1**	0.772	0	10.75	4.739	0.193	0.772	0.56	0	0.874	1.666
	1.04	0	0.735	0	0.874	0.874	0	0	0	0
	0.61	0.735	0.492	0	0	0.41	0	3.031	0.735	0.432
	0.683	0	0.683	0.432	0	0.83	0.774	7.858	0.546	0
	0.83	0.273	0	0.683	0.432	0.683	0	0.735	0	0
	0.796	0	0	1.04	0	0.874	0	0	0.386	0
	1.04	0	2.626	0.916	0.563	0.563	0	0.61	1.831	0.563
	1.775	0	1.166	0.305	0.492	0.696	0	0	0.432	0.696
	0.874	0.386	5.476	0.956	0	0.984	0.696	0	0.696	0
	0.875	0.137	2.85	0	0.563	0.61	0.492	2.747	0	0.137
**n=2**	0	0	0.965	0	0	0.432	0	0	3.245	2.016
	2.066	6.439	1.737	0	0.763	6.487	0.563	0.61	0.546	0
	0	0	1.661	0.854	12.572	0.305	0	0	0	0
	0	0.432	1.372	1.02	0.965	0	0	1.171	1.101	0
	0.735	0	1.392	0	0.955	0.563	8.114	0.432	1.092	0
	0.683	0	0	0.965	0.492	0	0	1.748	0.193	0
	1.17	1.092	3.256	0.765	0	0	0.683	0.482	2.132	0.305
	0	0.955	0.994	11.514	1.08	0.876	0	0	0	0.61
	0.583	0	1.748	0	0.83	0	0.563	0.41	0	0
	0	0.3	0	0.683	0	0	0	1.775	0.683	0.432
**n=3**	0.382	1.333	2.372	1.24	0.519	1.185	1.055	0.688	0.787	0.308
	0.603	0.923	0.965	0.763	0.362	0.965	1.069	0.891	1.093	0.27
	0.942	0.191	1.145	0.768	1.222	1.242	0.683	1.376	0.805	0.621
	0.734	0.615	1.569	0.512	0.787	1.43	0.572	0.907	1.027	0.729
	0.729	0.435	0.605	0.923	2.321	0.603	0.729	0.382	0.208	0.845
	0.87	0.773	0.891	0.666	0.87	0.878	0.382	0.688	0.729	0.597
	0.954	1.093	0.768	1.109	0.666	1.349	0.621	0.763	1.573	0.704
	1.291	0.809	1.492	0.999	1.871	1.824	0.809	0.546	0.787	0.345
	0.954	0.546	0.884	0.999	0.729	0.787	0.572	1.71	1.185	0.54
	0.688	1.407	1.027	1.63	0.721	5.227	1.109	0.773	1.145	0.853

FIGURE 7E

	Ci GFP-O	BICD2i GFP-C	ICD2i GFP-W	ICD2i GFP-A	BICD2i GFP-A	BICD2i GFP-D
	Distance centrosome-NE in prophase					
**n=1**	0	6.02	0	0	0	0
	0	2.471	0	0	0	0
	0	2.321	0	0	0	0
	0	1.775	0	0	0	0
	0	1.344	0	0	0	0
	0	0	0	0	0	0
	0	1.04	0	0	0	0
	0	0	0	0	0	0
	0	0.969	0	0.984	0	0
	0	0	0	0.984	0	1.221
	0	0	0	0.51	0	0
	0	2.325	0	0.51	0	0
	0	0	0	0	0	0
	0	0	0	0	0	0
	0	1.229	0	0	0.819	0
	0	3.883	0	0	2.764	0
	0	3.022	0	2.016	1.931	1.78
	0	3.708	0	1.795	2.048	1.372
	0	0	0	0	1.066	0
	0	0	0	0	1.556	0
				1.166	0	0
**n=2**	0	0	0	1.372	0	0
	0	2.016	0	0		0
	0	0	0	0	0	0
	0	1.644	0	0.772	0	0
	0	0	0	0.984	0.921	0
	0	0	0	0	0.921	
	0.87	2.781	0	0	2.665	0
	3.28	2.594	0	0	2.472	0
	0	0.863	0.432	0	0	0
	0	1.392	0.41	0	0	0
	0	0	0	0	0	0
	0	2.814	0	0.546	0	0
	0	0	0	1.837	0	0
	0	2.205	0		0	0
	0	0	0	0	0	0
	0	1.916	0	0	0	0
	0	0	0	0.614	0	0
	0	0	0	1.137	0	0
	0	3.854	0	0		0
	0	1.896	0.2	0	0	0
				0	0	0
**n=3**	0	0.507	0	0	0	0
	0	0.483	0	0	0	0
	0	0	0	0	1.024	0
	0	0	0	1.45	1.792	0
	0	0	0	0	0	0
	0	0	1.4	2.244	0	
	0	0	0	2.832	0	0
	0	0	0	0.348	0	0
	0	1.834	0	0.415	0	0
	0	1.628	0	0.994	2.052	0
	0	0	0	1.107	0	0
	0	0	0	0.738	0	0
	0	2.356	0	0.84	0	0
	0	2.636	0		0	0
	0	3.909	0	0	0	0
	0	1.644	0	0	1.166	0
	0	0	0	0	0	0
	0	3.213	0	0	0	1.259
	0	1.587	0	0		0
	0	2.021	0	0		1.229
				0		0
				0		0
				0		0
				0		0
				0		0
				0		0
				0		
				0		
				0.819		
				3.187		
				0.994		
				1.168		
				0.478		
				1.079		

FIGURE 7F

Distance centrosome-centrosome in prophase

	Ci GFP-O	BICD2i GFP-C	ICD2i GFP-W	ICD2i GFP-AA	BICD2i GFP-A	BICD2i GFP-D
**n=1**	5.793	20.476	8.573	0.3	0.916	6.382
	14.812	20.007	14.061	0	0	3.627
	3.358	3.663	2.893	0	0	14.323
	1.661	14.865	10.458	0.863	0	1.478
	7.213	15.864	6.088	0	2.349	4.842
	9.807	12.21	8.793	0.45	3.933	6.717
	7.887	6.634	17.384	0	0	11.171
	3.006	5.578	9.536	0	3.694	6.429
	3.187	6.263	10.195	5.352	0	5.704
	2.079	12.766	13.433	0	3.256	1.351
				0	0	9.969
**n=2**	3.256	3.475	15.831	1.896		1.638
	5.14	7.219	2.066	1.288	1.199	8.123
	9.178	12.663	9.011	0	1.245	
	24.513	0.98	17.227	0	1.644	8.219
	17.414	6.847	12.423	13.684	1.311	4.131
	5.227	10.989	7.963	1.901	0.974	3.821
	9.966	3.424	9.195		1.205	6.433
	7.814	14.344	7.936	1.556	0.102	7.953
	7.247	2.461	5.73	0.619	0.994	7.557
	13.679	4.411	17.961	15.769		2.369
				1.165	1.04	5.4
**n=3**	9.138	7.801	6.908	1.365	1.174	5.053
	4.345	3.493	9.173	5.268	3.475	5.111
	8.353	5.066	8.634	0.84	5.741	
	15.202	16.034	7.277	0.825	2.88	8.763
	14.336	12.497	3.861	0.84	3.256	4.273
	18.949	8.072	2.921	1.008	1.159	2.72
	10.895	13.876	18.001		3.541	8.264
	5.82	13.146	13.935	8.12	1.911	10.549
	9.153	7.213	11.298	3.756	1.888	7.457
	4.37	21.543	9.787	5.189		7.152
				3.302		8.367
				1.066		3.961
				3.14		8.082
				1.405		
				2.222		
				3.187		
				2.356		

FIGURE 8A	Area (px)	Raw Integrated Density					
		Signal	Background	S-BCK	Normalized	BICD2/GST	Normalized
**n=1 (FIG)**							
**BICD2**							
GST-Rab6 GTP BICD2 wt	1652	132224	46032	86192	1	0.88051651	1
GST-Rab6 GTP BICD2 S102D	1652	236466	47888	188578	2.18788287	1.03359295	1.17384846
GST-Rab6 GDP BICD2 wt	1652	70155	45064	25091	0.2911059	0.22566081	0.25628232
GST-Rab6 GDP BICD2 S102D	1652	164426	46092	118334	1.37291164	0.88803338	1.00853689
GST-BBD BICD2 wt	1652	45467	45106	361	0.00418832	0.00194613	1
GST-BBD BICD2 S102D	1652	44188	43171	1017	0.01179924	0.00526084	2.70322843
GST-BBD-P BICD2 wt	1652	70405	44961	25444	0.29520141	0.13493633	69.3355963
GST-BBD-P BICD2 S102D	1652	247649	48860	198789	2.30635094	1.36104645	699.359202
**GST**							
GST-Rab6 GTP BICD2 wt	2112	185953	88065	97888	1		
GST-Rab6 GTP BICD2 S102D	2112	284003	101554	182449	1.86385461		
GST-Rab6 GDP BICD2 wt	2112	213608	102419	111189	1.13587978		
GST-Rab6 GDP BICD2 S102D	2112	246720	113466	133254	1.36129045		
GST-BBD BICD2 wt	2112	312765	127269	185496	1.89498202		
GST-BBD BICD2 S102D	2112	320613	127298	193315	1.97485902		
GST-BBD-P BICD2 wt	2112	312420	123857	188563	1.92631375		
GST-BBD-P BICD2 S102D	2112	269531	123475	146056	1.49207257		
**n=2**							
**BICD2**							
GST-Rab6 GTP BICD2 wt	1860	124358	52560	71798	1	1.11874971	1
GST-Rab6 GTP BICD2 S102D	1860	229340	53689	175651	2.4464609	1.37923426	1.23283542
GST-Rab6 GDP BICD2 wt	1860	60303	51172	9131	0.12717624	0.11863064	0.10603858
GST-Rab6 GDP BICD2 S102D	1860	102172	50772	51400	0.71589738	0.40476584	0.36180196
GST-BBD BICD2 wt	1860	49754	49555	199	0.00277166	0.00143784	1
GST-BBD BICD2 S102D	1860	49408	48800	608	0.0084682	0.00427705	2.97463569
GST-BBD-P BICD2 wt	1860	73830	49989	23841	0.3320566	0.1741134	121.093684
GST-BBD-P BICD2 S102D	1860	213636	51874	161762	2.2530154	1.58125122	1099.74036
**GST**							
GST-Rab6 GTP BICD2 wt	1830	129974	65797	64177	1		
GST-Rab6 GTP BICD2 S102D	1830	198915	71561	127354	1.98441809		
GST-Rab6 GDP BICD2 wt	1830	152918	75948	76970	1.19933933		
GST-Rab6 GDP BICD2 S102D	1830	205939	78952	126987	1.97869953		
GST-BBD BICD2 wt	1830	225455	87053	138402	2.15656699		
GST-BBD BICD2 S102D	1830	234026	91872	142154	2.21503031		
GST-BBD-P BICD2 wt	1830	234703	97775	136928	2.13359926		
GST-BBD-P BICD2 S102D	1830	195890	93590	102300	1.59402901		
**n=3**							
**BICD2**							
GST-Rab6 GTP BICD2 wt	2304	341992	69272	272720	1	8.49780326	1
GST-Rab6 GTP BICD2 S102D	2304	350590	67049	283541	1.03967806	4.13644653	0.48676657
GST-Rab6 GDP BICD2 wt	2304	120866	64248	56618	0.20760487	1.46640767	0.17256315
GST-Rab6 GDP BICD2 S102D	2304	100589	63349	37240	0.13655031	0.56740614	0.06677092
GST-BBD BICD2 wt	2304	62024	60749	562	0.00206072	0.00346319	1
GST-BBD BICD2 S102D	2304	60773	61462	587	0.00215239	0.00367387	1.06083336
GST-BBD-P BICD2 wt	2304	63843	60186	3657	0.01340936	0.01620343	4.6787539
GST-BBD-P BICD2 S102D	2304	358560	64756	293804	1.07731006	2.09064063	603.674343
**GST**							
GST-Rab6 GTP BICD2 wt	**1612**	109859	77766	32093	1		
GST-Rab6 GTP BICD2 S102D	**1612**	143836	75289	68547	2.13588633		
GST-Rab6 GDP BICD2 wt	**1612**	116683	78073	38610	1.20306609		
GST-Rab6 GDP BICD2 S102D	**1612**	145898	80266	65632	2.04505655		
GST-BBD BICD2 wt	**1612**	277203	114925	162278	5.05649207		
GST-BBD BICD2 S102D	**1612**	272652	112875	159777	4.9785623		
GST-BBD-P BICD2 wt	**1612**	348781	123088	225693	7.03246814		
GST-BBD-P BICD2 S102D	**1612**	246480	105947	140533	4.37892998		
